# Correction: Complex polymorphisms in endocytosis genes suggest alpha-cyclodextrin as a treatment for breast cancer

**DOI:** 10.1371/journal.pone.0214826

**Published:** 2019-03-28

**Authors:** Knut M. Wittkowski, Christina Dadurian, Martin P. Seybold, Han Sang Kim, Ayuko Hoshino, David Lyden

In the “HPaCD is more effective than HPbCD against migration of breast cancer cells” subsection of the Results (page 14 of the PDF file), the first word of the third paragraph is incorrect. The cyclodextrin used in [72–74] was MβCD. In this study, the authors used HP cyclodextrins because of lower cytotoxicity. Thus, the second sentence in this paragraph is incorrect. The correct sentence is: To determine whether inhibition of migration is caused by cyclodextrins depleting cholesterol, as assumed previously, the published activity from wound healing experiments comparing MβCD against control was replicated using HPβCD, and complemented with novel activity results comparing HPαCD against control, both in MDA-MB 231 (ER-) and MCF-7 (ER+) human breast epithelial cell lines.
