# Supplementary material for: Complex polymorphisms in endocytosis genes suggest alpha-cyclodextrin as a treatment for breast cancer
Source: PLoS One. 2018 Jul 2;13(7):e0199012. doi: 10.1371/journal.pone.0199012 (PMC6028090; doi:10.1371/journal.pone.0199012)
Supplement: S1 File — Fig A. Manhattan Plots. Panels are CGEM, PGCS, and EPIC (from top to bottom). Dot size increases from single-SNP (black foreground) to six SNP diplotype (background). Color indicates information content (low: red; high: black). Values that were manually removed, because the diplotype spans LDBs or are higher than overlapping diplotypes are crossed out (white). First line of annotation indicates previous publication implicating this gene; second line implicates genes replicated in at least one of the other two populations. Fig B: QR-Plot of ssGWAS results by chromosome for CGEM. SNPs too far upstream ("-") or downstream ("+") to be considered related and genes with unknown function (e.g., LOC…, "?") are shown in gray among the results for individual chromosomes and are excluded from the summary plot. The "null" projection (blue) in the summary plot ends at the median among the endpoints of the convex projections for individual chromosomes. Genes to the right of the vertical blue line are above the cut-off for study-specific genome-wide significance.[13]. Fig C: QR-Plot of muGWAS results by chromosome for CGEM. In addition to the annotation used in Fig 2 of S1 File, genes whose significance relies entirely on a single SNP are marked in red ("$") and excluded from the summary plot. Fig D: String Analysis of Genes aGWS in ssGWAS that are Unrelated to Known Pathways. Connections: Co-Mentioned in PubMed abstracts (green), experimental/biochemical Data (pink), association in curated database (blue); Table A: Genes Involved in EEC identified in breast cancer. Bold: aGWS. *: from previous GWAS. Underlined: functionally related genes identified in the literature. †: implied. Table B: Top and replicated genes. Replicated genes (left column) and aGWS results are shown in bold, results below the level of support (row aGWS/2) shown in gray; BC Ref: selected references related to breast cancer; a: Top genes in ssGWAS; b: Top consecutive set of replicated genes by population; c: Ot [file pone.0199012.s001.pdf]

S1 Supplementary Material

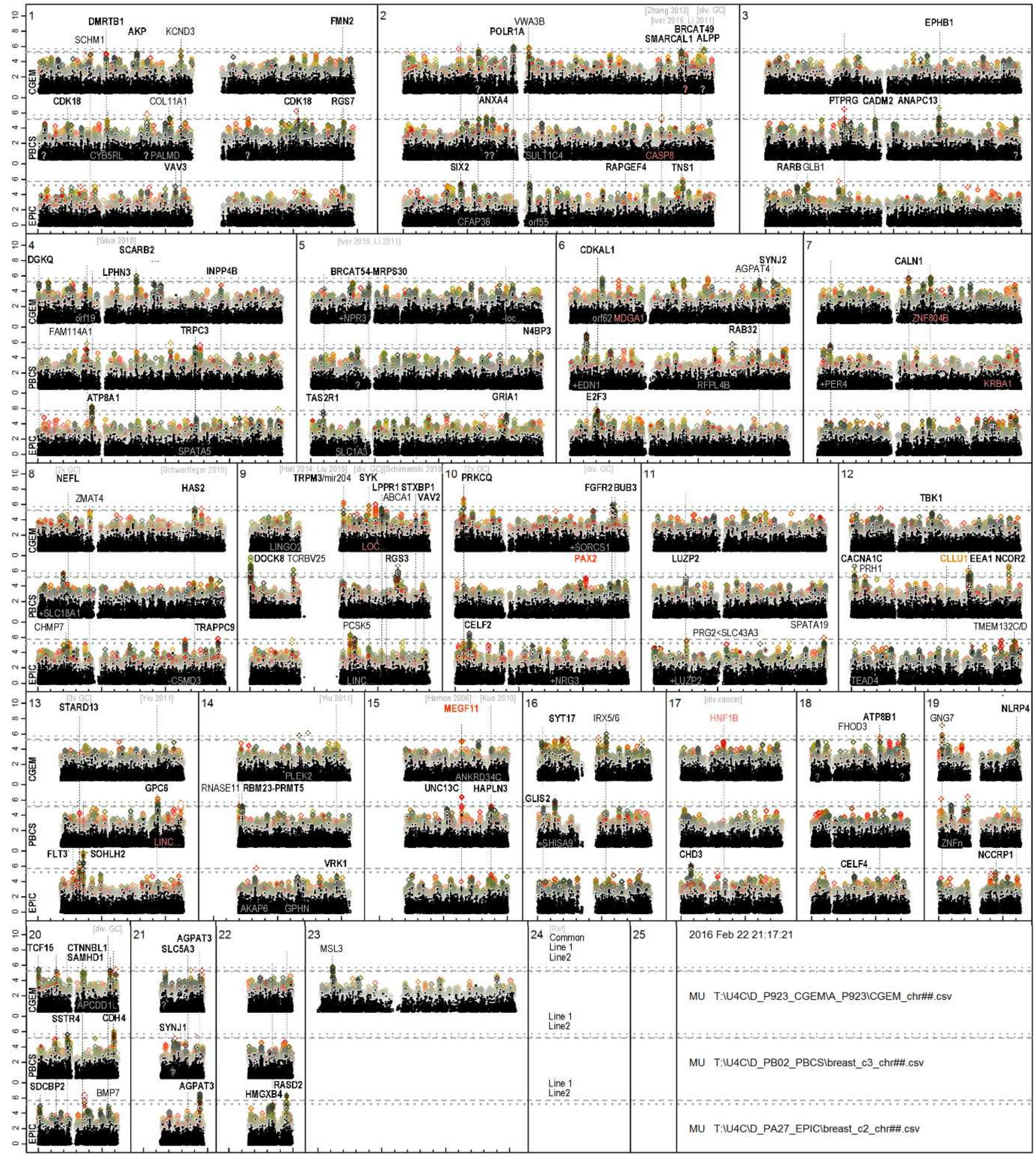

Fig A. Manhattan Plots. Panels are CGEM, PGCS, and EPIC (from top to bottom). Dot size increases from single-SNP (black foreground) to six SNP diplotype (background). Color indicates information content (low: red; high: black). Values that were manually removed, because the diplotype spans LDBs or are higher than overlapping diplotypes are crossed out (white). First line of annotation indicates previous publication implicating this gene; second line implicates genes replicated in at least one of the other two populations.

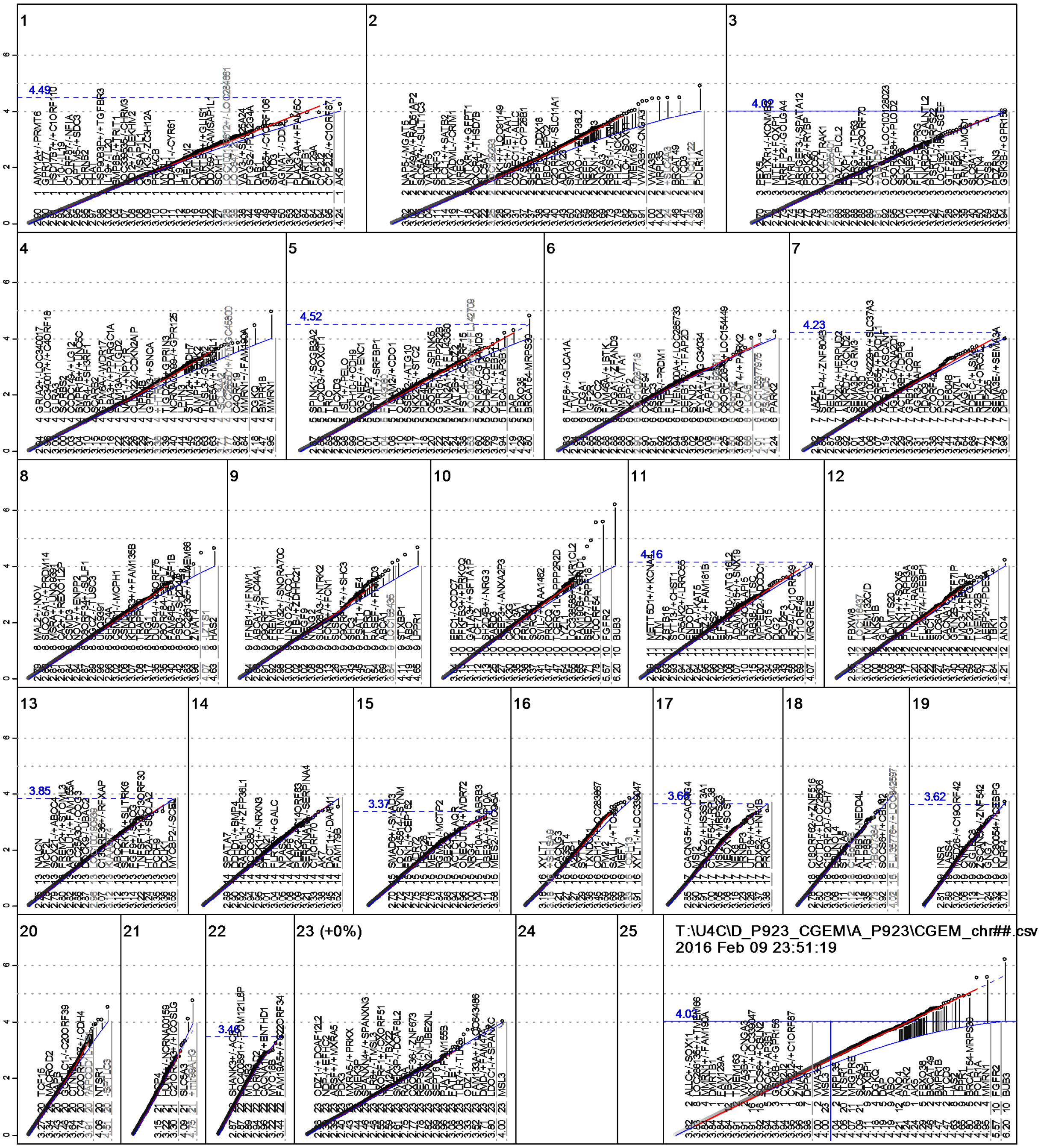

Fig B: QR-Plot of ssGWAS results by chromosome for CGEM. SNPs too far upstream ("-") or downstream ("+") to be considered related and genes with unknown function (e.g., LOC..., "?") are shown in gray among the results for individual chromosomes and are excluded from the summary plot. The "null" projection (blue) in the summary plot ends at the median among the endpoints of the convex projections for individual chromosomes. Genes to the right of the vertical blue line are above the cut-off for study-specific genome-wide significance.[13]

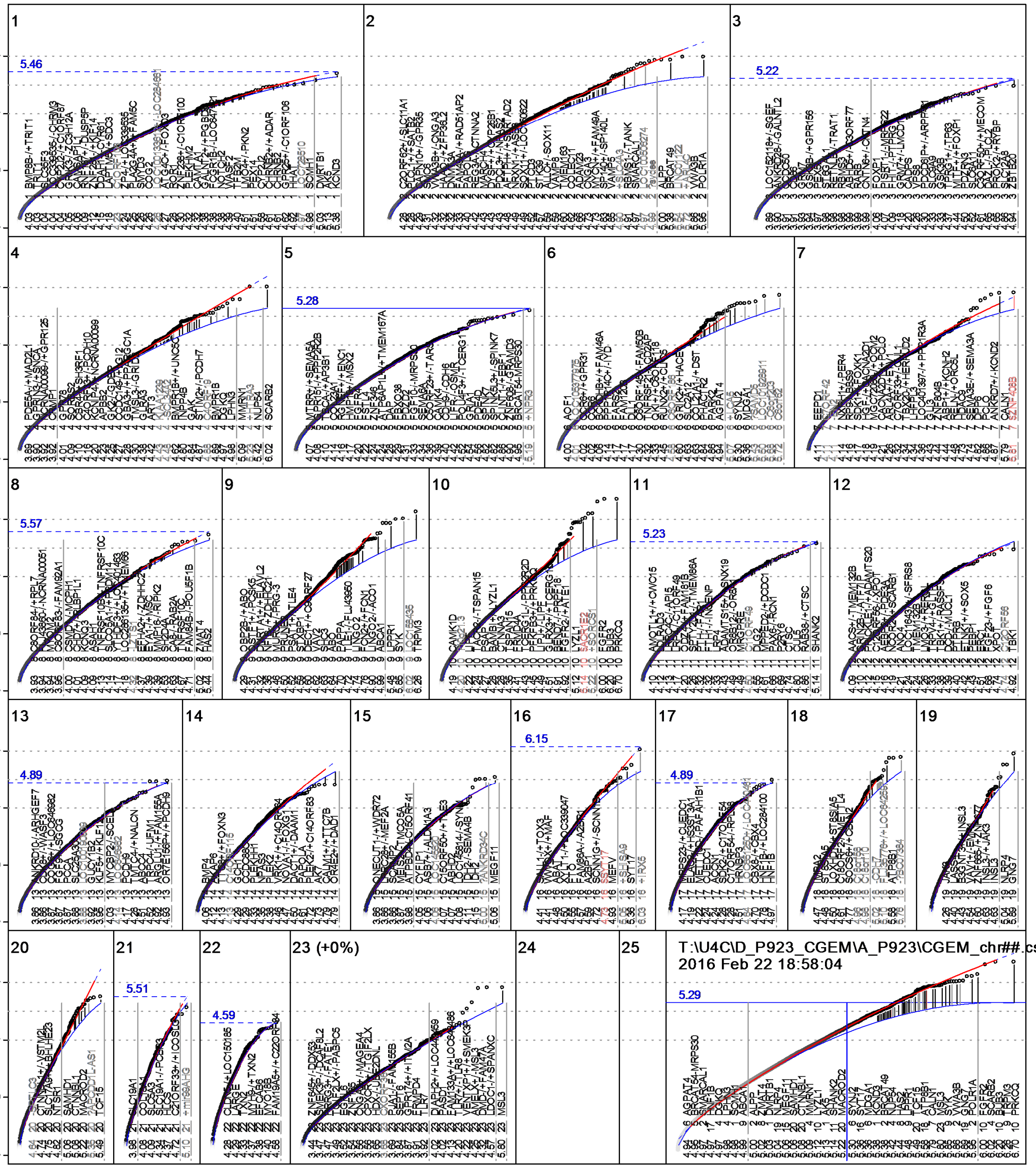

Fig C: QR-Plot of muGWAS results by chromosome for CGEM. In addition to the annotation used in S1 Fig 2, genes whose significance relies entirely on a single SNP are marked in red ("$") and excluded from the summary plot.

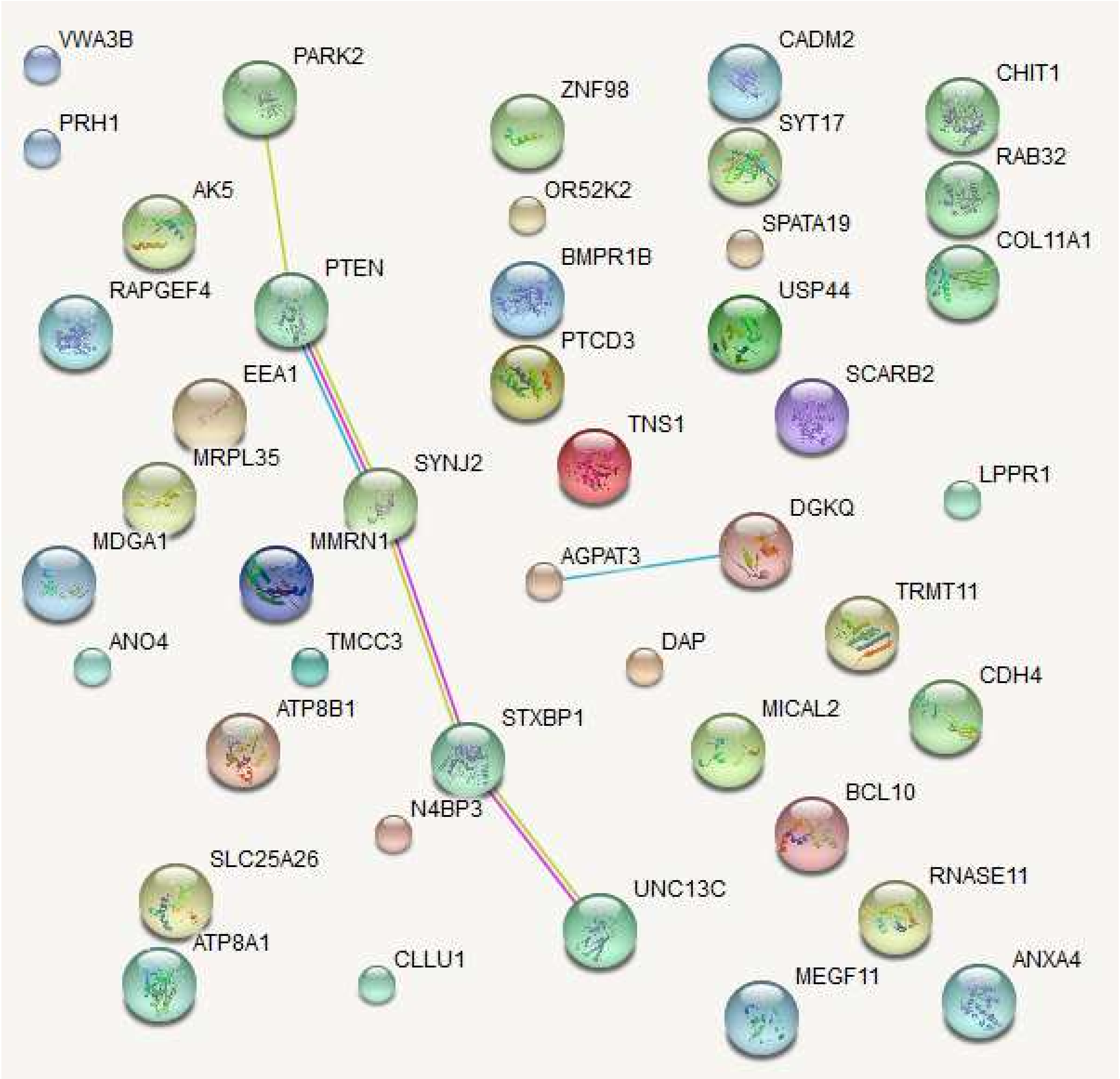

Fig D: String Analysis of Genes aGWS in ssGWAS that are Unrelated to Known Pathways. Connections: Co-Mentioned in PubMed abstracts (green), experimental/biochemical Data (pink), association in curated database (blue);

Table A: **Genes Involved in EEC identified in** breast cancer**.** Bold: aGWS. *: from previous GWAS. Underlined: functionally related genes identified in the literature. †: implied

Gene	Entrez gene	Len kB	Function	EEC Function	BC Reference
ATP8A1	10396	249	Increasing extracellular PC and PS enhances endocy-	[64, 166]	[167, 168]
ATP8B1	5205	85	tosis		
ANO4	12161	334	Ca+ dependent phospholipid scramblase	[169]	
ABCA1	19	147	Regulates cellular lipid efflux; upregulation ameliorates Huntington's disease.[170]	[171]	[172, 173]
AGPAT3	56894	122	converts lysophosphatidylinositol (LPI) into phosphati-	[174]	[175, 176]
AGPAT4	56895	144	dylinositol (PI)	hsa00564	
DGKQ	1609	15	Regenerates PI from DAG	hsa00564, hsa04070	[177]
LPPR1	54886	296	complexes with *LPPR3/4/5*, regulates PIS/CDIPT	[178]	[179] †
ASTN2	23245	990	regulates trafficking of *ASTN1*, during early clathrin-dependent endocytosis; binds AP-2 (*AP2xn*)	[67, 180, 181] hsa04144	?
TNS1	7145	144	controls cell polarization, migration and invasion binds a5b1 integrin during endocytosis	[182-184]	[185]
MEGF11	84465	358	In *C. elegans*, DYN-1 (*DNM1*) depends on the function of CED-1 (*MEGF10/11*)	[186] hsa04144 hsa04721	[173]
ABCA1	19	147	interacts with *MEGF10*	[171]	
SDCBP2	27111	19	Syndecans bind PI(4,5)P2 and are involved in both endo- and exocytosis.	[187, 188]	[189]
N4BP3	23138	13	*NEDD4* controls growth factor receptor endocytosis (*NEDD9* expression is assoc. with BC metastasis)	[190, 191] hsa04144	[191-193]
SYNJ2	8871	117	is recruited to the nascent clathrin coated pit	[194] hsa04070	[63]
NLRP4	147945	45	and *NLRP3* associate with *BECN1, a component of the PI3K complex that mediates vesicle trafficking*	[195-197]	[198]
PTENP1	11191	4	PI3K/PTEN and PI(3,4,5)P3 are involved in endocytosis and cancer	[199] hsa04070	[200]
VAV3	10451	394	*VAV* ... promote BCR endocytosis	[201, 202] hsa04666	[203]
*PDE4D**	5144	925	Binds *ARRB2* (fast recycling)	[204] hsa04144	[205]
EEA1	8411	157	binds to early endosomes in a Rab5 and PI(3)P dependent manner.	[206] hsa04144	
RAB32	10981	11	*RAB32/RAB38* interact AP-3 and with *LRRK2* (PARK8)	[207-209] hsa05012	[210]
SNX32*	254122	20	Sorting Nexin (late endosome), SNX-BAR retromer with other Vps17 orthologs SNX5/SNX6 interacts with VPS35	[211-213] hsa04144	[214]
SCARB2	950	55	required for maintenance of endo- and lysosomes, located in limiting membranes	[215] hsa04142	[216, 217]
GLB1	2720	101	Galactosidase Beta, related to Galectin 3 (*LGALS3*)	[218]	[219]
RAPGEF4	11069	317	GEF for *RAB1A/1B/2A*; involved in excocytosis through *RIMS2*	[220, 221]	[222]
UNC13C	440279	616	Interacting with each other and with PI(4,5)P2. In-	[223, 224]	[225]
STXBP1 (MUNC18)	6812		volved in docking/priming in exocytosis	hsa04721	
STXBP4*	252983	195	Prevents interaction between *STX4* and *VAMP2*,	[213] hsa04130,hsa04721	[226, 227]
ANXA4	307	84	Forms exocytotic complexes with *SYT1* and the *RAB3A* effector *RPH3A*.	[228, 229] hsa04721	[230, 231]
SYT17	51760	99	"B/K protein may play a role in exocytosis"	[232, 233]	[234]
PARK2	5071	1380	"Loss of parkin promotes ... endocytosis by accumulating CAV1"; *PARK2* binds AP-2 via arrestin	[235, 236] hsa04141	[237]
DNAJC1*	64215	247	ER membrane protein. *DNAJC* (Hsp40) controls release of proteins via *HSPA5* (BiP, *GRP78*); *DNAJC13* interacts with SNX-BAR	hsa04141	[50, 238]

Table B: Top and replicated genes. Replicated genes (left column) and aGWS results are shown in bold, results below the level of support (row aGWS/2) shown in gray; BC Ref: selected references related to breast cancer; a: Top genes in ssGWAS; b: Top consecutive set of replicated genes by population; c: Other replicated significant genes; d: Additional replicated genes – see Text for details.

Gene	Chr	CGEM			EPIC			PBCS			BC Ref
		s6	s1		s6	s1		s6	s1		
aGWS:		5.29	4.03		5.71	4.00		5.13	3.84		see also
aGWS/2		*2.56*	*2.02*		*2.86*	*2.00*		*2.57*	*1.92*		S1 Table 3
a: ***FGFR2***	10	**6.00***	**5.57***	rs1219648§	3.09	*1.23*	rs1631281	3.18	*1.82*	rs4752566	[239, 240]
BUB3	10	**6.20***	**6.20***	rs10510126§	*2.68*	*1.64*	rs2495771				[241]
MMRN1	4	**5.09***	**4.95***	rs12233759§							[242]
CHD3	17	*<2.0*	*1.20*	rs11078712	**6.13***	**5.66***	rs4791889	*2.47*	*1.06*	rs4321249	[243]
SOHLH2	13	*<2.0*	*1.58*	rs9593921	**8.58***	**5.42***	rs1928021				[244, 245]
DOCK8	9							**7.74***	**5.83***	rs943628	[246]
b: ***PRKCQ***	10	**6.70***	3.47	**rs661891**	3.32	*1.96*	**rs661891**	*<2.0*	*<1.0*		[35, 247]
		**5.47***	3.11	rs658230	5.26	3.87	rs591441	*<2.0*	*<1.0*		
TRPM3-mir204	9	**6.26***	3.68	rs1329776	4.13	2.20	rs1328153	4.52	3.78	rs7868074	[37, 248]
TRPC3[6)]	4	*<2.0*	*1.58*		*<2.0*	*1.58*		**5.35***	**4.86***	rs1918179	[249]
SCARB2	4	**6.02***	3.15	rs17001561				4.59	2.35	rs6846983	[216]
POLR1A	2	**5.95***	**4.89***	**rs10779967**§				3.55	2.74	**rs10779967**	[250]
AGPAT3[1)]	21	3.57	2.63	**rs8132053**	**6.59***	**4.73***	**rs8132053**	3.32	2.38	rs11089102	[176]
AGPAT4[1)]	6	4.94	3.56	rs9347493	3.88	2.62	rs3734462§				[175]
CELF2	10	4.05	2.04	rs4750033	**6.51***	4.10*	rs10490955	*2.97*	*1.87*	rs2224867	[251]
STARD13	13	*<2.0*	*1.50*	rs17078802	**6.48***	**4.56***	rs9596958	4.31	2.14	rs17078802	[38]
NCOR2	12	4.09	2.32	rs275769	3.00	*1.61*	rs7972214	**6.63***	3.35	rs10846667	[40]
CACNA1C	12	3.38	3.37	rs16927947	*2.71*	*1.82*	rs4765687	**6.59***	**4.95***	rs7311147	[41]
MEGF11	15	5.06	3.67	rs170781	4.01	3.31	rs333554	**6.39***	**4.07***	rs12903880	↔ ABCA1
GPC6	13	3.02	*1.95*	rs1333259	3.45	*1.80*	rs7324202	**6.22***	**4.82***	rs9561536	[252]
c: ***GNG7***	19	**5.89***	3.24	rs11880582	3.01	*1.95*	rs2058459	*2.26*	*1.77*	rs12462317	head/neck
VWA3B	2	**5.86***	4.00	rs6715989§	3.72	3.10	rs2309389				binds **β1** integrin
LPPR1[5)]	9	**5.48***	**4.40***	rs13284030	3.56	*1.76*	**rs10819896**	*2.46*	*1.78*	**rs10819896**	
DGKQ[5)]	4	4.97	**4.18***	**rs2290405**	<2.0	<1.0		2.88	*1.27*	**rs2290405**	[177]
PARK2	6	4.86	**4.24***	rs2023048	3.57	2.31	rs3798964	3.31	*1.41*	rs2097130	[237]
SYNJ2	6	**5.30***	3.02	rs2295894	*<2.0*	*<1.0*		4.03	2.33	rs750997	[63]
ATP8B1[3)]	18	**5.58***	3.35	rs317809	4.11	*1.94*	rs6566896				[167]
ATP8A1[3)]	4				**6.26***	**4.87***	rs10517048				[168]
STXBP1[7)]	9	4.09	**4.11***	rs2287116	*<2.0*	*<1.0*		*<2.0*	*<1.0*		[213]
UNC13C[7)]	15	3.49	*1.25*	rs2414299	3.08	2.27	rs884043	4.91	**4.41***	rs2414376	squamous cell
TNS1[4)]	2	3.30	2.60	rs6729308	**5.88***	**4.19***	rs1427675	*<2.0*	*<1.0*		[185]
PTENP1[4)]	9	*<2.0*	*<1.0*		*<2.0*	*<1.0*		**6.14***	3.81	rs1856201	*PTEN*
TRAPPC9	8	3.29	3.08	**rs6987302**	**5.84***	**4.36***	rs4289794	*2.33*	2.33	**rs6987302**	[253]
BMPR1B[2)]	4	4.89	**4.47***	rs1970801							[44]
BMP7[2)]	20	3.48	2.13	rs186659	4.84	**4.24***	rs186659	*<2.0*	*<1.0*		[254]
RAPGEF4	2	4.02	3.28	rs2060773	*2.61*	**4.22***	rs6727383				[255]
RGS3	9	3.49	2.64	rs664850	3.35	*1.82*	rs568557	**5.97***	**5.80***	rs670719	[256]
d: *ASTN2*	9	3.73	2.98	rs10983479	5.33	*1.93*	rs10121207	4.13	*1.55*	rs2225067	
PDE4D	5	4.56	3.36	rs6896121	4.46	*1.81*	rs40216	2.84	*1.17*	rs1423473	[257, 258]
VAV3	1	3.39	3.39	rs17019880	5.05	3.16	rs17019427	3.09	*1.80*	rs1887630	[203]
NLRP11/4/8	19	5.04	3.70	rs441827	4.59	2.50	rs11665811				[198]

[1)]1-Acylglycerol-3-Phosphate O-Acyltransferase paralogs, convert lysophosphatic acid (LPA) into PA, the second step in de novo phospholipid biosynthesis (hsa00564) [2)]Receptor-ligand pair (hsa04060) [3)]P-type ATPase paralogs, form flippase complex with *TMEM30A*, transport amino-phospholipids from the outer to the inner leaflet of membranes [4)]homologs, Cowden syndrome, [5)]pre/post PA, [6)]*TRPxn,* [$]quoted in dbGaP from [7]

Table C: Known regulators of integrin cycling in breast cancer. Top: all genes related to breast cancer and endocytosis,[46, 259] *: integrin trafficking genes related to breast cancer.[48]

	Function in CM-EEC	Integrin relevance		breast cancer phenotype
**TNK2**	Ack1, endoced by *TNK2*, amplifies Arp2/3 dynamics by phosphorylating cortactin[260]	Ack1 is involved in integrin β1-mediated cell adhesion[261]	β1	activation correlates with metastatic state[262]
**CTTN**	Cortactin binds dynamin to actin,[259]	Inhibition of β1 signaling attenuates phosphorylation of cortactin[263]	β1	Cortactin, paxillin, and β1-integrin facilitate metastases in breast cancer[263]
**ARHGEF7 (COOL1, β-PIX)**	prevents Cbl from ubiquitinating [264]	Cbl ubiquitinates α5 integrin and suppresses[265] Rap1 activation of integrins[266]	a5	functions in cell migration[GC] GIT1/bPix complexes are essential for integrin-mediated cell motility[267]
**SPRY2**	sequesters c-Cbl[268]	β1 expression depends on SPRY2[269]	β1	Spry1 promotes triple-negative breast cancer [270]
**HAX1***	assists with formation of clathrin-coated vesicles,[46]	required for endocytosis of integrin αvβ6[271]	αvβ6	increases avβ6-dependent invasion[271]
**GIPC***		stimulates internalization of α5β1 in EEs.[272]	α5β1	regulates breast cancer cell invasion [273]
**NUMB***	localized to CCP[274]	required for endocytosis of β1 integrins[275, 276]	β1/3	1)
**DAB2***	binds integrins/*APP*/*LRP8*, *MYO6*, PI(4,5)P_2, clathrin [277]	is required for endocytosis of β1 integrins[275, 276]	β1/3	required to initiate collective invasion in breast cancer[278]
**ACAP1**	interacts with *ARF6*	recycles β1 integrins [82]	β1	upregulated in breast cancer [79]
**IQSEC1 (GP100)**	GEF for ARP on membranes that contain PI(4.5)P2[hsa04144]	depletion inhibits β1 integrin endocytosis[275]	β1	breast cancer specific invasion and metastasis[279]
**HIP1**	interacts with AP2A1/2[hsa0516]	required for integrin turnover [280]		upregulates epithelial-mesenchymal transition in breast cancer[281]
**NDRG1**	RAB4A effector	RAB4 controls short-loop recycling[282]		metastasis suppressor downregulated in breast cancer
**RAB5***	master regulator of early endosome dynamics[283]		β1	is necessary for formation of invadosomes[284]
**RAB11FIP1* (RCP)**	RAB11 effector[285]	drives long-loop integrin α5β1 recycling[286]	α5β1	promotes breast cancer[287] overexpressed in BC
**MYO10***	is a PI(3)P effector[288]	transports integrins to the filopodial tip[289, 290]	β1	required for invadopodial formation[291, 292]
**GBA**	breaks down glucocerebroside (GC) in the lysosome.[293]	GBA increases expression of β1 integrin[294]	β1	GBA upregulated in breast cancer,[295] but downregulated in PD[296, 297]
**LAMP1**	"Lysosomal Associated Membrane Protein"	delayed acidification promotes recycling via CLIC3[182]	α5β1	LAMP1 colocalizes with Rab11 in the LY[182]
**P SEN2**	regulates LY acidification[298]	(see LAMP1/2)		R62H and R71W mutations in breast cancer[299]
**CTSD**	Lysosomal Aspartyl Peptidase	protein degradation in the lysosome		Prognostic indicator of breast cancer metastases[300]
**RAB25***	involved in long-loop recycling.	directs active α5β1 to the lysosome[45]	α5β1	promotes tumor cell invasion [301, 302]
**CLIC3***	Intracellular chloride channel	Promotes α5β1 recycling form LE/LY (w/RAB25).[45]	α5β1	Drives cancer progression (with RAB25) [45]n

1) NUMB reduces BC cell migration also by degrading **NOTCH**;[303] NUMB also interacts with MDM2. MDM2 also induces EMT in breast cancer cells by upregulating Snail.[304]

Knockdown of STXBPx 4 also substantially inhibited β1-integrin recycling in human monocytes.[305]

7. Hunter DJ, Kraft P, Jacobs KB, Cox DG, Yeager M, Hankinson SE, et al. A genome-wide association study identifies alleles in FGFR2 associated with risk of sporadic postmenopausal breast cancer. Nature Genet. 2007;39(7):870-4. doi: 10.1038/ng2075. PubMed PMID: PMC3493132.
13. Wittkowski KM, Sonakya V, Bigio B, Tonn MK, Shic F, Ascano M, et al. A novel computational biostatistics approach implies impaired dephosphorylation of growth factor receptors as associated with severity of autism. Transl Psychiatry. 2014;4:e354. doi: 10.1038/tp.2013.124. PubMed Central PMCID: PMC3905234.
35. Zafar A, Wu F, Hardy K, Li J, Tu WJ, McCuaig R, et al. Chromatinized protein kinase C-theta directly regulates inducible genes in epithelial to mesenchymal transition and breast cancer stem cells. Mol Cell Biol. 2014;34(16):2961-80. Epub 2014/06/04. doi: 10.1128/mcb.01693-13. PubMed PMID: 24891615; PubMed Central PMCID: PMC4135602.
37. Hall DP, Cost NG, Hegde S, Kellner E, Mikhaylova O, Stratton Y, et al. TRPM3 and miR-204 establish a regulatory circuit that controls oncogenic autophagy in clear cell renal cell carcinoma. Cancer cell. 2014;26(5):738-53. Epub 2014/12/18. doi: 10.1016/j.ccell.2014.09.015. PubMed PMID: 25517751; PubMed Central PMCID: PMC4269832.
38. Hanna S, Khalil B, Nasrallah A, Saykali BA, Sobh R, Nasser S, et al. StarD13 is a tumor suppressor in breast cancer that regulates cell motility and invasion. Int J Oncol. 2014;44(5):1499-511. Epub 2014/03/15. doi: 10.3892/ijo.2014.2330. PubMed PMID: 24627003; PubMed Central PMCID: PMC4027929.
40. Zhang L, Gong C, Lau SL, Yang N, Wong OG, Cheung AN, et al. SpliceArray profiling of breast cancer reveals a novel variant of NCOR2/SMRT that is associated with tamoxifen resistance and control of ERalpha transcriptional activity. Cancer Res. 2013;73(1):246-55. Epub 2012/11/03. doi: 10.1158/0008-5472.can-12-2241. PubMed PMID: 23117886.
41. Wang CY, Lai MD, Phan NN, Sun Z, Lin YC. Meta-Analysis of Public Microarray Datasets Reveals Voltage-Gated Calcium Gene Signatures in Clinical Cancer Patients. PLoS One. 2015;10(7):e0125766. Epub 2015/07/07. doi: 10.1371/journal.pone.0125766. PubMed PMID: 26147197; PubMed Central PMCID: PMC4493072.
44. Yan H, Zhu S, Song C, Liu N, Kang J. Bone morphogenetic protein (BMP) signaling regulates mitotic checkpoint protein levels in human breast cancer cells. Cell Signal. 2012;24(4):961-8. Epub 2012/01/12. doi: 10.1016/j.cellsig.2011.12.019. PubMed PMID: 22234345.
45. Dozynkiewicz MA, Jamieson NB, Macpherson I, Grindlay J, van den Berghe PV, von Thun A, et al. Rab25 and CLIC3 collaborate to promote integrin recycling from late endosomes/lysosomes and drive cancer progression. Dev Cell. 2012;22(1):131-45. Epub 2011/12/27. doi: 10.1016/j.devcel.2011.11.008. PubMed PMID: 22197222; PubMed Central PMCID: PMC3507630.
46. Mosesson Y, Mills GB, Yarden Y. Derailed endocytosis: an emerging feature of cancer. Nat Rev Cancer. 2008;8(11):835-50. doi: http://www.nature.com/nrc/journal/v8/n11/suppinfo/nrc2521_S1.html.
48. De Franceschi N, Hamidi H, Alanko J, Sahgal P, Ivaska J. Integrin traffic - the update. J Cell Sci. 2015;128(5):839-52. Epub 2015/02/11. doi: 10.1242/jcs.161653. PubMed PMID: 25663697; PubMed Central PMCID: PMC4342575.
50. Michailidou K, Hall P, Gonzalez-Neira A, Ghoussaini M, Dennis J, Milne RL, et al. Large-scale genotyping identifies 41 new loci associated with breast cancer risk. Nat Genet. 2013;45(4):353-61, 61e1-2. Epub 2013/03/29. doi: 10.1038/ng.2563. PubMed PMID: 23535729; PubMed Central PMCID: PMC3771688.
63. Ben-Chetrit N, Chetrit D, Russell R, Korner C, Mancini M, Abdul-Hai A, et al. Synaptojanin 2 is a druggable mediator of metastasis and the gene is overexpressed and amplified in breast cancer. Sci Signal. 2015;8(360):ra7. Epub 2015/01/22. doi: 10.1126/scisignal.2005537. PubMed PMID: 25605973.
64. Farge E, Ojcius DM, Subtil A, Dautry-Varsat A. Enhancement of endocytosis due to aminophospholipid transport across the plasma membrane of living cells. Am J Physiol. 1999;276(3 Pt 1):C725-33. Epub 1999/03/10. PubMed PMID: 10070001.
67. Wilson PM, Fryer RH, Fang Y, Hatten ME. Astn2, A Novel Member of the Astrotactin Gene Family, Regulates the Trafficking of ASTN1 during Glial-Guided Neuronal Migration. The Journal of Neuroscience. 2010;30(25):8529-40. doi: 10.1523/jneurosci.0032-10.2010.

79. Hoffman JD, Graff RE, Emami NC, Tai CG, Passarelli MN, Hu D, et al. Cis-eQTL-based trans-ethnic meta-analysis reveals novel genes associated with breast cancer risk. PLoS Genet. 2017;13(3):e1006690. Epub 2017/04/01. doi: 10.1371/journal.pgen.1006690. PubMed PMID: 28362817; PubMed Central PMCID: PMCPMC5391966.
82. Li J, Ballif BA, Powelka AM, Dai J, Gygi SP, Hsu VW. Phosphorylation of ACAP1 by Akt regulates the stimulation-dependent recycling of integrin beta1 to control cell migration. Dev Cell. 2005;9(5):663-73. Epub 2005/11/01. doi: 10.1016/j.devcel.2005.09.012. PubMed PMID: 16256741.
166. Levano K, Sobocki T, Jayman F, Debata PR, Sobocka MB, Banerjee P. A genetic strategy involving a glycosyltransferase promoter and a lipid translocating enzyme to eliminate cancer cells. Glycoconj J. 2009;26(6):739-48. Epub 2009/03/14. doi: 10.1007/s10719-009-9233-1. PubMed PMID: 19283471.
167. da Costa A, Lenze D, Hummel M, Kohn B, Gruber AD, Klopfleisch R. Identification of six potential markers for the detection of circulating canine mammary tumour cells in the peripheral blood identified by microarray analysis. J Comp Pathol. 2012;146(2-3):143-51. Epub 2011/07/26. doi: 10.1016/j.jcpa.2011.06.004. PubMed PMID: 21783201.
168. Sjöblom T, Jones S, Wood LD, Parsons DW, Lin J, Barber TD, et al. The Consensus Coding Sequences of Human Breast and Colorectal Cancers. Science. 2006;314(5797):268-74. doi: 10.1126/science.1133427.
169. Picollo A, Malvezzi M, Accardi A. TMEM16 proteins: unknown structure and confusing functions. J Mol Biol. 2015;427(1):94-105. Epub 2014/12/03. doi: 10.1016/j.jmb.2014.09.028
S0022-2836(14)00547-6 [pii]. PubMed PMID: 25451786; PubMed Central PMCID: PMC4277903.
170. Valenza M, Marullo M, Di Paolo E, Cesana E, Zuccato C, Biella G, et al. Disruption of astrocyte-neuron cholesterol cross talk affects neuronal function in Huntington's disease. Cell Death Differ. 2015;22(4):690-702. Epub 2014/10/11. doi: 10.1038/cdd.2014.162. PubMed PMID: 25301063; PubMed Central PMCID: PMCPMC4356339.
171. Hamon Y, Trompier D, Ma Z, Venegas V, Pophillat M, Mignotte V, et al. Cooperation between Engulfment Receptors: The Case of ABCA1 and MEGF10. PLoS One. 2006;1(1):e120. doi: 10.1371/journal.pone.0000120.
172. Zhao W, Prijic S, Urban BC, Tisza MJ, Zuo Y, Li L, et al. Candidate Antimetastasis Drugs Suppress the Metastatic Capacity of Breast Cancer Cells by Reducing Membrane Fluidity. Cancer Res. 2016;76(7):2037-49. Epub 2016/01/31. doi: 10.1158/0008-5472.can-15-1970. PubMed PMID: 26825169.
173. Schimanski S, Wild PJ, Treeck O, Horn F, Sigruener A, Rudolph C, et al. Expression of the lipid transporters ABCA3 and ABCA1 is diminished in human breast cancer tissue. Horm Metab Res. 2010;42(2):102-9. Epub 2009/11/11. doi: 10.1055/s-0029-1241859. PubMed PMID: 19902402.
174. Bradley RM, Marvyn PM, Aristizabal Henao JJ, Mardian EB, George S, Aucoin MG, et al. Acylglycerophosphate acyltransferase 4 (AGPAT4) is a mitochondrial lysophosphatidic acid acyltransferase that regulates brain phosphatidylcholine, phosphatidylethanolamine, and phosphatidylinositol levels. Biochimica et Biophysica Acta (BBA) - Molecular and Cell Biology of Lipids. 2015;1851(12):1566-76. doi: http://dx.doi.org/10.1016/j.bbalip.2015.09.005.
175. Sahay D, Leblanc R, Grunewald TG, Ambatipudi S, Ribeiro J, Clezardin P, et al. The LPA1/ZEB1/miR-21-activation pathway regulates metastasis in basal breast cancer. Oncotarget. 2015;6(24):20604-20. Epub 2015/06/23. doi: 10.18632/oncotarget.3774. PubMed PMID: 26098771; PubMed Central PMCID: PMC4653029.
176. Hopkins MM, Zhang Z, Liu Z, Meier KE. Eicosopentaneoic Acid and Other Free Fatty Acid Receptor Agonists Inhibit Lysophosphatidic Acid- and Epidermal Growth Factor-Induced Proliferation of Human Breast Cancer Cells. J Clin Med. 2016;5(2). Epub 2016/01/29. doi: 10.3390/jcm5020016. PubMed PMID: 26821052; PubMed Central PMCID: PMC4773772.
177. Filigheddu N, Cutrupi S, Porporato PE, Riboni F, Baldanzi G, Chianale F, et al. Diacylglycerol kinase is required for HGF-induced invasiveness and anchorage-independent growth of MDA-MB-231 breast cancer cells. Anticancer Res. 2007;27(3B):1489-92. Epub 2007/06/29. PubMed PMID: 17595766.
178. Yu P, Agbaegbu C, Malide DA, Wu X, Katagiri Y, Hammer JA, et al. Cooperative interactions of LPPR family members in membrane localization and alteration of cellular morphology. Journal of Cell Science. 2015;128(17):3210-22.

179. Ngan E, Northey JJ, Brown CM, Ursini-Siegel J, Siegel PM. A complex containing LPP and alpha-actinin mediates TGFbeta-induced migration and invasion of ErbB2-expressing breast cancer cells. J Cell Sci. 2013;126(Pt 9):1981-91. Epub 2013/03/01. doi: 10.1242/jcs.118315. PubMed PMID: 23447672; PubMed Central PMCID: PMC3791827.
180. Solecki DJ. Sticky situations: recent advances in control of cell adhesion during neuronal migration. Curr Opin Neurobiol. 2012;22(5):791-8. Epub 2012/05/09. doi: 10.1016/j.conb.2012.04.010 S0959-4388(12)00068-2 [pii]. PubMed PMID: 22560352; PubMed Central PMCID: PMC3551464.
181. Kawauchi T. Cell adhesion and its endocytic regulation in cell migration during neural development and cancer metastasis. Int J Mol Sci. 2012;13(4):4564-90. Epub 2012/05/19. doi: 10.3390/ijms13044564. PubMed PMID: 22605996; PubMed Central PMCID: PMC3344232.
182. Rainero E, Howe JD, Caswell PT, Jamieson NB, Anderson K, Critchley DR, et al. Ligand-Occupied Integrin Internalization Links Nutrient Signaling to Invasive Migration. Cell Rep. 2015;10:398-413. Epub 2015/01/21. doi: 10.1016/j.celrep.2014.12.037. PubMed PMID: 25600874.
183. McCleverty CJ, Lin DC, Liddington RC. Structure of the PTB domain of tensin1 and a model for its recruitment to fibrillar adhesions. Protein Science : A Publication of the Protein Society. 2007;16(6):1223-9. doi: 10.1110/ps.072798707. PubMed PMID: PMC2206669.
184. Burghel GJ, Lin W-Y, Whitehouse H, Brock I, Hammond D, Bury J, et al. Identification of Candidate Driver Genes in Common Focal Chromosomal Aberrations of Microsatellite Stable Colorectal Cancer. PLoS One. 2013;8(12):e83859. doi: 10.1371/journal.pone.0083859.
185. Hall EH, Daugherty AE, Choi CK, Horwitz AF, Brautigan DL. Tensin1 requires protein phosphatase-1alpha in addition to RhoGAP DLC-1 to control cell polarization, migration, and invasion. J Biol Chem. 2009;284(50):34713-22. Epub 2009/10/15. doi: 10.1074/jbc.M109.059592. PubMed PMID: 19826001; PubMed Central PMCID: PMC2787334.
186. Shen Q, He B, Lu N, Conradt B, Grant BD, Zhou Z. Phagocytic receptor signaling regulates clathrin and epsin-mediated cytoskeletal remodeling during apoptotic cell engulfment in C. elegans. Development. 2013;140(15):3230-43. Epub 2013/07/19. doi: 10.1242/dev.093732. PubMed PMID: 23861060; PubMed Central PMCID: PMC3931732.
187. Baietti MF, Zhang Z, Mortier E, Melchior A, Degeest G, Geeraerts A, et al. Syndecan-syntenin-ALIX regulates the biogenesis of exosomes. Nat Cell Biol. 2012;14(7):677-85. Epub 2012/06/05. doi: 10.1038/ncb2502. PubMed PMID: 22660413.
188. Hurley JH, Odorizzi G. Get on the exosome bus with ALIX. Nat Cell Biol. 2012;14(7):654-5. Epub 2012/06/30. doi: 10.1038/ncb2530. PubMed PMID: 22743708.
189. Yang Y, Hong Q, Shi P, Liu Z, Luo J, Shao Z. Elevated expression of syntenin in breast cancer is correlated with lymph node metastasis and poor patient survival. Breast Cancer Res. 2013;15(3):R50. Epub 2013/06/22. doi: 10.1186/bcr3442. PubMed PMID: 23786877; PubMed Central PMCID: PMC4053163.
190. Persaud A, Alberts P, Hayes M, Guettler S, Clarke I, Sicheri F, et al. Nedd4-1 binds and ubiquitylates activated FGFR1 to control its endocytosis and function. EMBO J. 2011;30(16):3259-73. Epub 2011/07/19. doi: 10.1038/emboj.2011.234. PubMed PMID: 21765395; PubMed Central PMCID: PMC3160656.
191. Jung S, Li C, Jeong D, Lee S, Ohk J, Park M, et al. Oncogenic function of p34SEI-1 via NEDD41mediated PTEN ubiquitination/degradation and activation of the PI3K/AKT pathway. Int J Oncol. 2013;43(5):1587-95. Epub 2013/08/24. doi: 10.3892/ijo.2013.2064. PubMed PMID: 23970032.
192. Minn AJ, Gupta GP, Siegel PM, Bos PD, Shu W, Giri DD, et al. Genes that mediate breast cancer metastasis to lung. Nature. 2005;436(7050):518-24. Epub 2005/07/29. doi: 10.1038/nature03799. PubMed PMID: 16049480; PubMed Central PMCID: PMC1283098.
193. Liao CJ, Chi HC, Tsai CY, Chen CD, Wu SM, Tseng YH, et al. A novel small-form NEDD4 regulates cell invasiveness and apoptosis to promote tumor metastasis. Oncotarget. 2015;6(11):9341-54. Epub 2015/04/01. doi: 10.18632/oncotarget.3322. PubMed PMID: 25823820; PubMed Central PMCID: PMC4496221.
194. Schmid SL, Mettlen M. Cell biology: Lipid switches and traffic control. Nature. 2013;499(7457):161-2. doi: 10.1038/nature12408.
195. Jounai N, Kobiyama K, Shiina M, Ogata K, Ishii KJ, Takeshita F. NLRP4 negatively regulates autophagic processes through an association with beclin1. J Immunol. 2011;186(3):1646-55. Epub 2011/01/07. doi: 10.4049/jimmunol.1001654. PubMed PMID: 21209283.

196. Zhang Y, Sauler M, Shinn AS, Gong H, Haslip M, Shan P, et al. Endothelial PINK1 Mediates the Protective Effects of NLRP3 Deficiency during Lethal Oxidant Injury. The Journal of Immunology. 2014;192(11):5296-304. doi: 10.4049/jimmunol.1400653.
197. Rohatgi RA, Shaw LM. An autophagy-independent function for Beclin 1 in cancer. Mol Cell Oncol. 2016;3(1). Epub 2016/03/22. doi: 10.1080/23723556.2015.1030539. PubMed PMID: 26998512; PubMed Central PMCID: PMC4792009.
198. Zhiyu W, Wang N, Wang Q, Peng C, Zhang J, Liu P, et al. The inflammasome: an emerging therapeutic oncotarget for cancer prevention. Oncotarget. 2016;7(31):50766. Epub 2016/05/22. doi: 10.18632/oncotarget.9391. PubMed PMID: 27206676.
199. Erneux C, Ghosh S, Ramos AR, Edimo WE. New Functions of the Inositol Polyphosphate 5-Phosphatases in Cancer. Curr Pharm Des. 2016;22(16):2309-14. Epub 2016/02/27. PubMed PMID: 26916021.
200. Zhang H-Y, Liang F, Jia Z-L, Song S-T, Jiang Z-F. PTEN mutation, methylation and expression in breast cancer patients. Oncology Letters. 2013;6(1):161-8. doi: 10.3892/ol.2013.1331. PubMed PMID: PMC3742525.
201. Inabe K, Ishiai M, Scharenberg AM, Freshney N, Downward J, Kurosaki T. Vav3 modulates B cell receptor responses by regulating phosphoinositide 3-kinase activation. J Exp Med. 2002;195(2):189-200. Epub 2002/01/24. PubMed PMID: 11805146; PubMed Central PMCID: PMC2193613.
202. Malhotra S, Kovats S, Zhang W, Coggeshall KM. Vav and Rac Activation in B Cell Antigen Receptor Endocytosis Involves Vav Recruitment to the Adapter Protein LAB. The Journal of Biological Chemistry. 2009;284(52):36202-12. doi: 10.1074/jbc.M109.040089. PubMed PMID: PMC2794736.
203. Chen XIN, Chen SI, Liu X-A, Zhou W-B, Ma R-R, Chen LIN. Vav3 oncogene is upregulated and a poor prognostic factor in breast cancer patients. Oncology Letters. 2015;9(5):2143-8. doi: 10.3892/ol.2015.3004. PubMed PMID: PMC4467222.
204. Haddad SA, Ruiz-Narvaez EA, Haiman CA, Sucheston-Campbell LE, Bensen JT, Zhu Q, et al. An exome-wide analysis of low frequency and rare variants in relation to risk of breast cancer in African American Women: the AMBER Consortium. Carcinogenesis. 2016. Epub 2016/06/09. doi: 10.1093/carcin/bgw067. PubMed PMID: 27267999.
205. Lin D-C, Xu L, Ding L-W, Sharma A, Liu L-Z, Yang H, et al. Genomic and functional characterizations of phosphodiesterase subtype 4D in human cancers. Proc Natl Acad Sci USA. 2013;110(15):6109-14. doi: 10.1073/pnas.1218206110. PubMed PMID: PMC3625360.
206. Pfeffer SR. Motivating endosome motility. Nat Cell Biol. 1999;1(6):E145-E7.
207. Waschbusch D, Michels H, Strassheim S, Ossendorf E, Kessler D, Gloeckner CJ, et al. LRRK2 transport is regulated by its novel interacting partner Rab32. PLoS One. 2014;9(10):e111632. Epub 2014/11/02. doi: 10.1371/journal.pone.0111632. PubMed PMID: 25360523; PubMed Central PMCID: PMC4216093.
208. Bultema JJ, Ambrosio AL, Burek CL, Di Pietro SM. BLOC-2, AP-3, and AP-1 proteins function in concert with Rab38 and Rab32 proteins to mediate protein trafficking to lysosome-related organelles. J Biol Chem. 2012;287(23):19550-63. Epub 2012/04/19. doi: 10.1074/jbc.M112.351908. PubMed PMID: 22511774; PubMed Central PMCID: PMC3365991.
209. Hesketh GG, Perez-Dorado I, Jackson LP, Wartosch L, Schafer IB, Gray SR, et al. VARP is recruited on to endosomes by direct interaction with retromer, where together they function in export to the cell surface. Dev Cell. 2014;29(5):591-606. Epub 2014/05/27. doi: 10.1016/j.devcel.2014.04.010. PubMed PMID: 24856514; PubMed Central PMCID: PMC4059916.
210. Agalliu I, San Luciano M, Mirelman A, Giladi N, Waro B, Aasly J, et al. Higher frequency of certain cancers in LRRK2 G2019S mutation carriers with Parkinson disease: a pooled analysis. JAMA Neurol. 2015;72(1):58-65. Epub 2014/11/18. doi: 10.1001/jamaneurol.2014.1973. PubMed PMID: 25401981; PubMed Central PMCID: PMC4366130.
211. Wang X, Huang T, Bu G, Xu H. Dysregulation of protein trafficking in neurodegeneration. Molecular Neurodegeneration. 2014;9(1):1-9. doi: 10.1186/1750-1326-9-31.
212. van Weering JR, Verkade P, Cullen PJ. SNX-BAR-mediated endosome tubulation is co-ordinated with endosome maturation. Traffic. 2012;13(1):94-107. Epub 2011/10/07. doi: 10.1111/j.1600-0854.2011.01297.x. PubMed PMID: 21973056.
213. Zhang QY, Tan MS, Yu JT, Tan L. The Role of Retromer in Alzheimer's Disease. Mol Neurobiol. 2015. Epub 2015/07/29. doi: 10.1007/s12035-015-9366-0. PubMed PMID: 26215837.

214. Rivera J, Megias D, Bravo J. Sorting nexin 6 interacts with breast cancer metastasis suppressor-1 and promotes transcriptional repression. J Cell Biochem. 2010;111(6):1464-72. Epub 2010/09/11. doi: 10.1002/jcb.22874. PubMed PMID: 20830743.
215. Gonzalez A, Valeiras M, Sidransky E, Tayebi N. Lysosomal integral membrane protein-2: a new player in lysosome-related pathology. Mol Genet Metab. 2014;111(2):84-91. Epub 2014/01/07. doi: 10.1016/j.ymgme.2013.12.005. PubMed PMID: 24389070; PubMed Central PMCID: PMC3924958.
216. Nishimura Y, Yoshioka K, Bernard O, Bereczky B, Itoh K. A role of LIM kinase 1/cofilin pathway in regulating endocytic trafficking of EGF receptor in human breast cancer cells. Histochem Cell Biol. 2006;126(5):627-38. Epub 2006/06/10. doi: 10.1007/s00418-006-0198-x. PubMed PMID: 16763828.
217. Nishimura Y, Itoh K, Yoshioka K, Tokuda K, Himeno M. Overexpression of ROCK in human breast cancer cells: evidence that ROCK activity mediates intracellular membrane traffic of lysosomes. Pathol Oncol Res. 2003;9(2):83-95. Epub 2003/07/15. doi: Paor.2003.9.2.0083. PubMed PMID: 12858212.
218. Ahmed H, AlSadek DM. Galectin-3 as a Potential Target to Prevent Cancer Metastasis. Clin Med Insights Oncol. 2015;9:113-21. Epub 2015/12/08. doi: 10.4137/cmo.s29462. PubMed PMID: 26640395; PubMed Central PMCID: PMC4662425.
219. O'Reilly EA, Gubbins L, Sharma S, Tully R, Guang MH, Weiner-Gorzel K, et al. The fate of chemoresistance in triple negative breast cancer (TNBC). BBA Clin. 2015;3:257-75. Epub 2015/12/18. doi: 10.1016/j.bbacli.2015.03.003. PubMed PMID: 26676166; PubMed Central PMCID: PMC4661576.
220. Parnell E, Palmer TM, Yarwood SJ. The future of EPAC-targeted therapies: agonism versus antagonism. Trends Pharmacol Sci. 2015;36(4):203-14. Epub 2015/03/07. doi: 10.1016/j.tips.2015.02.003. PubMed PMID: 25744542; PubMed Central PMCID: PMC4392396.
221. Almahariq M, Tsalkova T, Mei FC, Chen H, Zhou J, Sastry SK, et al. A novel EPAC-specific inhibitor suppresses pancreatic cancer cell migration and invasion. Mol Pharmacol. 2013;83(1):122-8. Epub 2012/10/16. doi: 10.1124/mol.112.080689. PubMed PMID: 23066090; PubMed Central PMCID: PMC3533471.
222. Jiang HL, Sun HF, Gao SP, Li LD, Hu X, Wu J, et al. Loss of RAB1B promotes triple-negative breast cancer metastasis by activating TGF-beta/SMAD signaling. Oncotarget. 2015;6(18):16352-65. Epub 2015/05/15. doi: 10.18632/oncotarget.3877. PubMed PMID: 25970785; PubMed Central PMCID: PMC4599274.
223. Betz A, Okamoto M, Benseler F, Brose N. Direct interaction of the rat unc-13 homologue Munc13-1 with the N terminus of syntaxin. J Biol Chem. 1997;272(4):2520-6. Epub 1997/01/24. PubMed PMID: 8999968.
224. Martin TF. PI(4,5)P(2)-binding effector proteins for vesicle exocytosis. Biochim Biophys Acta. 2015;1851(6):785-93. Epub 2014/10/05. doi: 10.1016/j.bbalip.2014.09.017. PubMed PMID: 25280637; PubMed Central PMCID: PMC4380529.
225. Fernandez-Nogueira P, Bragado P, Almendro V, Ametller E, Rios J, Choudhury S, et al. Differential expression of neurogenes among breast cancer subtypes identifies high risk patients. Oncotarget. 2016;7(5):5313-26. Epub 2015/12/18. doi: 10.18632/oncotarget.6543. PubMed PMID: 26673618; PubMed Central PMCID: PMC4868688.
226. Antoniou AC, Beesley J, McGuffog L, Sinilnikova OM, Healey S, Neuhausen SL, et al. Common breast cancer susceptibility alleles and the risk of breast cancer for BRCA1 and BRCA2 mutation carriers: implications for risk prediction. Cancer Res. 2010;70(23):9742-54. Epub 2010/12/02. doi: 10.1158/0008-5472.can-10-1907. PubMed PMID: 21118973; PubMed Central PMCID: PMC2999830.
227. Day P, Riggs KA, Hasan N, Corbin D, Humphrey D, Hu C. Syntaxins 3 and 4 mediate vesicular trafficking of alpha5beta1 and alpha3beta1 integrins and cancer cell migration. Int J Oncol. 2011;39(4):863-71. Epub 2011/07/02. doi: 10.3892/ijo.2011.1101. PubMed PMID: 21720706.
228. Lizarbe MA, Barrasa JI, Olmo N, Gavilanes F, Turnay J. Annexin-phospholipid interactions. Functional implications. Int J Mol Sci. 2013;14(2):2652-83. Epub 2013/01/30. doi: 10.3390/ijms14022652. PubMed PMID: 23358253; PubMed Central PMCID: PMC3588008.
229. Willshaw A, Grant K, Yan J, Rockliffe N, Ambavarapu S, Burdyga G, et al. Identification of a novel protein complex containing annexin A4, rabphilin and synaptotagmin. FEBS Lett. 2004;559(1-3):13-21. Epub 2004/02/13. doi: 10.1016/s0014-5793(03)01513-8. PubMed PMID: 14960300.
230. Wei B, Guo C, Liu S, Sun MZ. Annexin A4 and cancer. Clin Chim Acta. 2015;447:72-8. Epub 2015/06/07. doi: 10.1016/j.cca.2015.05.016. PubMed PMID: 26048190.
231. Yao H, Sun C, Hu Z, Wang W. The role of annexin A4 in cancer. Front Biosci (Landmark Ed). 2016;21:949-57. Epub 2016/04/23. PubMed PMID: 27100483.

232. Chin H, Choi SH, Jang YS, Cho SM, Kim HS, Lee JH, et al. Protein kinase A-dependent phosphorylation of B/K protein. Exp Mol Med. 2006;38(2):144-52. Epub 2006/05/05. doi: 10.1038/emm.2006.18. PubMed PMID: 16672768.
233. Fukuda M. The role of synaptotagmin and synaptotagmin-like protein (Slp) in regulated exocytosis. Madame Curie Regulated Database [Internet]. 2013.
234. Weng L, Ziliak D, Im HK, Gamazon ER, Philips S, Nguyen AT, et al. Genome-wide discovery of genetic variants affecting tamoxifen sensitivity and their clinical and functional validation. Annals of Oncology. 2013. doi: 10.1093/annonc/mdt125.
235. Cha SH, Choi YR, Heo CH, Kang SJ, Joe EH, Jou I, et al. Loss of parkin promotes lipid rafts-dependent endocytosis through accumulating caveolin-1: implications for Parkinson's disease. Mol Neurodegener. 2015;10:63. Epub 2015/12/03. doi: 10.1186/s13024-015-0060-5. PubMed PMID: 26627850; PubMed Central PMCID: PMC4666086.
236. Ahmed MR, Zhan X, Song X, Kook S, Gurevich VV, Gurevich EV. Ubiquitin ligase parkin promotes Mdm2-arrestin interaction but inhibits arrestin ubiquitination. Biochemistry. 2011;50(18):3749-63. Epub 2011/04/07. doi: 10.1021/bi200175q. PubMed PMID: 21466165; PubMed Central PMCID: PMC3091828.
237. Wang H, Liu B, Zhang C, Peng G, Liu M, Li D, et al. Parkin regulates paclitaxel sensitivity in breast cancer via a microtubule-dependent mechanism. J Pathol. 2009;218(1):76-85. Epub 2009/02/14. doi: 10.1002/path.2512. PubMed PMID: 19214989.
238. Chen C-L, Hou W-H, Liu I-H, Hsiao G, Huang SS, Huang JS. Inhibitors of clathrin-dependent endocytosis enhance TGFβ signaling and responses. Journal of Cell Science. 2009;122(11):1863-71. doi: 10.1242/jcs.038729.
239. Campbell TM, Castro MA, de Santiago I, Fletcher MN, Halim S, Prathalingam R, et al. FGFR2 risk SNPs confer breast cancer risk by augmenting oestrogen responsiveness. Carcinogenesis. 2016;37(8):741-50. Epub 2016/05/29. doi: 10.1093/carcin/bgw065. PubMed PMID: 27236187; PubMed Central PMCID: PMC4967216.
240. Valta MP, Hentunen T, Qu Q, Valve EM, Harjula A, Seppanen JA, et al. Regulation of osteoblast differentiation: a novel function for fibroblast growth factor 8. Endocrinology. 2006;147(5):2171-82. Epub 2006/01/28. doi: 10.1210/en.2005-1502. PubMed PMID: 16439448.
241. Mukherjee A, Joseph C, Craze M, Chrysanthou E, Ellis IO. The role of BUB and CDC proteins in low-grade breast cancers. Lancet. 2015;385 Suppl 1:S72. Epub 2015/08/28. doi: 10.1016/s0140-6736(15)60387-7. PubMed PMID: 26312894.
242. Zhao H, Langerod A, Ji Y, Nowels KW, Nesland JM, Tibshirani R, et al. Different gene expression patterns in invasive lobular and ductal carcinomas of the breast. Mol Biol Cell. 2004;15(6):2523-36. Epub 2004/03/23. doi: 10.1091/mbc.E03-11-0786. PubMed PMID: 15034139; PubMed Central PMCID: PMC420079.
243. Fujita N, Jaye DL, Kajita M, Geigerman C, Moreno CS, Wade PA. MTA3, a Mi-2/NuRD complex subunit, regulates an invasive growth pathway in breast cancer. Cell. 2003;113(2):207-19. Epub 2003/04/23. PubMed PMID: 12705869.
244. Ji S, Zhang W, Zhang X, Hao C, Hao A, Gao Q, et al. Sohlh2 suppresses epithelial to mesenchymal transition in breast cancer via downregulation of IL-8. Oncotarget. 2016. Epub 2016/07/08. doi: 10.18632/oncotarget.10355. PubMed PMID: 27384482.
245. Laurin M, Huber J, Pelletier A, Houalla T, Park M, Fukui Y, et al. Rac-specific guanine nucleotide exchange factor DOCK1 is a critical regulator of HER2-mediated breast cancer metastasis. Proc Natl Acad Sci USA. 2013;110(18):7434-9. doi: 10.1073/pnas.1213050110. PubMed PMID: PMC3645577.
246. Takahashi K, Kohno T, Ajima R, Sasaki H, Minna JD, Fujiwara T, et al. Homozygous deletion and reduced expression of the DOCK8 gene in human lung cancer. Int J Oncol. 2006;28(2):321-8. Epub 2006/01/05. PubMed PMID: 16391785.
247. Irie HY, Halstead-Nussloch G, Ito K. Abstract P4-05-10: PRKCQ, a novel protein kinase C preferentially expressed in triple negative breast cancer, drives oncogenic growth, survival and migration. Cancer Research. 2015;75(9 Supplement):P4-05-10. doi: 10.1158/1538-7445.sabcs14-p4-05-10.
248. Li W, Jin X, Zhang Q, Zhang G, Deng X, Ma L. Decreased expression of miR-204 is associated with poor prognosis in patients with breast cancer. International Journal of Clinical and Experimental Pathology. 2014;7(6):3287-92. PubMed PMID: PMC4097245.

249. Aydar E, Yeo S, Djamgoz M, Palmer C. Abnormal expression, localization and interaction of canonical transient receptor potential ion channels in human breast cancer cell lines and tissues: a potential target for breast cancer diagnosis and therapy. Cancer Cell Int. 2009;9:23. Epub 2009/08/20. doi: 10.1186/1475-2867-9-23. PubMed PMID: 19689790; PubMed Central PMCID: PMC2737535.
250. Li LY, Chen H, Hsieh YH, Wang YN, Chu HJ, Chen YH, et al. Nuclear ErbB2 enhances translation and cell growth by activating transcription of ribosomal RNA genes. Cancer Res. 2011;71(12):4269-79. Epub 2011/05/11. doi: 10.1158/0008-5472.can-10-3504. PubMed PMID: 21555369; PubMed Central PMCID: PMC3117049.
251. Mukhopadhyay D, Jung J, Murmu N, Houchen CW, Dieckgraefe BK, Anant S. CUGBP2 plays a critical role in apoptosis of breast cancer cells in response to genotoxic injury. Ann N Y Acad Sci. 2003;1010:504-9. Epub 2004/03/23. PubMed PMID: 15033780.
252. Yiu GK, Kaunisto A, Chin YR, Toker A. NFAT promotes carcinoma invasive migration through glypican-6. Biochem J. 2011;440(1):157-66. Epub 2011/08/30. doi: 10.1042/bj20110530. PubMed PMID: 21871017; PubMed Central PMCID: PMC3204871.
253. Chaligne R, Popova T, Mendoza-Parra MA, Saleem MA, Gentien D, Ban K, et al. The inactive X chromosome is epigenetically unstable and transcriptionally labile in breast cancer. Genome Res. 2015;25(4):488-503. Epub 2015/02/06. doi: 10.1101/gr.185926.114. PubMed PMID: 25653311; PubMed Central PMCID: PMC4381521.
254. Slattery ML, John EM, Torres-Mejia G, Herrick JS, Giuliano AR, Baumgartner KB, et al. Genetic variation in bone morphogenetic proteins and breast cancer risk in hispanic and non-hispanic white women: The breast cancer health disparities study. Int J Cancer. 2013;132(12):2928-39. Epub 2012/11/28. doi: 10.1002/ijc.27960. PubMed PMID: 23180569; PubMed Central PMCID: PMC3653321.
255. Pongor L, Kormos M, Hatzis C, Pusztai L, Szabo A, Gyorffy B. A genome-wide approach to link genotype to clinical outcome by utilizing next generation sequencing and gene chip data of 6,697 breast cancer patients. Genome Med. 2015;7:104. Epub 2015/10/18. doi: 10.1186/s13073-015-0228-1. PubMed PMID: 26474971; PubMed Central PMCID: PMC4609150.
256. Ooe A, Kato K, Noguchi S. Possible involvement of CCT5, RGS3, and YKT6 genes up-regulated in p53-mutated tumors in resistance to docetaxel in human breast cancers. Breast Cancer Res Treat. 2007;101(3):305-15. Epub 2006/07/06. doi: 10.1007/s10549-006-9293-x. PubMed PMID: 16821082.
257. Lin DC, Xu L, Ding LW, Sharma A, Liu LZ, Yang H, et al. Genomic and functional characterizations of phosphodiesterase subtype 4D in human cancers. Proc Natl Acad Sci U S A. 2013;110(15):6109-14. Epub 2013/03/29. doi: 10.1073/pnas.1218206110
1218206110 [pii]. PubMed PMID: 23536305; PubMed Central PMCID: PMC3625360.
258. Natrajan R, Mackay A, Lambros MB, Weigelt B, Wilkerson PM, Manie E, et al. A whole-genome massively parallel sequencing analysis of BRCA1 mutant oestrogen receptor-negative and -positive breast cancers. J Pathol. 2012;227(1):29-41. Epub 2012/03/01. doi: 10.1002/path.4003. PubMed PMID: 22362584.
259. Mellman I, Yarden Y. Endocytosis and Cancer. Cold Spring Harbor Perspectives in Biology. 2013;5(12). doi: 10.1101/cshperspect.a016949.
260. Kelley LC, Weed SA. Cortactin is a substrate of activated Cdc42-associated kinase 1 (ACK1) during ligand-induced epidermal growth factor receptor downregulation. PLoS One. 2012;7(8):e44363. Epub 2012/09/07. doi: 10.1371/journal.pone.0044363. PubMed PMID: 22952966; PubMed Central PMCID: PMC3431376.
261. Prieto-Echague V, Miller WT. Regulation of ack-family nonreceptor tyrosine kinases. J Signal Transduct. 2011;2011:742372. Epub 2011/06/04. doi: 10.1155/2011/742372. PubMed PMID: 21637378; PubMed Central PMCID: PMC3101793.
262. Mahajan K, Lawrence HR, Lawrence NJ, Mahajan NP. ACK1 tyrosine kinase interacts with histone demethylase KDM3A to regulate the mammary tumor oncogene HOXA1. J Biol Chem. 2014;289(41):28179-91. Epub 2014/08/26. doi: 10.1074/jbc.M114.584425. PubMed PMID: 25148682; PubMed Central PMCID: PMC4192474.
263. McFarlane S, McFarlane C, Montgomery N, Hill A, Waugh DJ. CD44-mediated activation of alpha5beta1-integrin, cortactin and paxillin signaling underpins adhesion of basal-like breast cancer cells to endothelium and fibronectin-enriched matrices. Oncotarget. 2015;6(34):36762-73. Epub 2015/10/09. doi: 10.18632/oncotarget.5461. PubMed PMID: 26447611; PubMed Central PMCID: PMC4742209.

264. Feng Q, Baird D, Peng X, Wang J, Ly T, Guan JL, et al. Cool-1 functions as an essential regulatory node for EGF receptor- and Src-mediated cell growth. Nat Cell Biol. 2006;8(9):945-56. Epub 2006/08/08. doi: 10.1038/ncb1453. PubMed PMID: 16892055.
265. Huang C. Roles of E3 ubiquitin ligases in cell adhesion and migration. Cell Adh Migr. 2010;4(1):10-8. Epub 2009/12/17. PubMed PMID: 20009572; PubMed Central PMCID: PMC2852552.
266. Bos JL, de Bruyn K, Enserink J, Kuiperij B, Rangarajan S, Rehmann H, et al. The role of Rap1 in integrin-mediated cell adhesion. Biochem Soc Trans. 2003;31(Pt 1):83-6. Epub 2003/01/28. doi: 10.1042/. PubMed PMID: 12546659.
267. Jones NP, Katan M. Role of phospholipase Cgamma1 in cell spreading requires association with a beta-Pix/GIT1-containing complex, leading to activation of Cdc42 and Rac1. Mol Cell Biol. 2007;27(16):5790-805. Epub 2007/06/15. doi: 10.1128/mcb.00778-07. PubMed PMID: 17562871; PubMed Central PMCID: PMC1952113.
268. Edwin F, Patel TB. A novel role of Sprouty 2 in regulating cellular apoptosis. J Biol Chem. 2008;283(6):3181-90. Epub 2007/12/12. doi: 10.1074/jbc.M706567200. PubMed PMID: 18070883; PubMed Central PMCID: PMC2527238.
269. Grassian AR, Schafer ZT, Brugge JS. ErbB2 stabilizes epidermal growth factor receptor (EGFR) expression via Erk and Sprouty2 in extracellular matrix-detached cells. J Biol Chem. 2011;286(1):79-90. Epub 2010/10/20. doi: 10.1074/jbc.M110.169821. PubMed PMID: 20956544; PubMed Central PMCID: PMC3013038.
270. He Q, Jing H, Liaw L, Gower L, Vary C, Hua S, et al. Suppression of Spry1 inhibits triple-negative breast cancer malignancy by decreasing EGF/EGFR mediated mesenchymal phenotype. Sci Rep. 2016;6:23216. Epub 2016/03/16. doi: 10.1038/srep23216. PubMed PMID: 26976794; PubMed Central PMCID: PMC4791662.
271. Ramsay AG, Keppler MD, Jazayeri M, Thomas GJ, Parsons M, Violette S, et al. HS1-associated protein X-1 regulates carcinoma cell migration and invasion via clathrin-mediated endocytosis of integrin alphavbeta6. Cancer Res. 2007;67(11):5275-84. Epub 2007/06/05. doi: 10.1158/0008-5472.can-07-0318. PubMed PMID: 17545607.
272. Valdembri D, Caswell PT, Anderson KI, Schwarz JP, Konig I, Astanina E, et al. Neuropilin-1/GIPC1 signaling regulates alpha5beta1 integrin traffic and function in endothelial cells. PLoS Biol. 2009;7(1):e25. Epub 2009/01/30. doi: 10.1371/journal.pbio.1000025. PubMed PMID: 19175293; PubMed Central PMCID: PMC2631072.
273. Wu D, Haruta A, Wei Q. GIPC1 interacts with MyoGEF and promotes MDA-MB-231 breast cancer cell invasion. J Biol Chem. 2010;285(37):28643-50. Epub 2010/07/17. doi: 10.1074/jbc.M110.107649. PubMed PMID: 20634288; PubMed Central PMCID: PMC2937890.
274. Nishimura T, Kaibuchi K. Numb controls integrin endocytosis for directional cell migration with aPKC and PAR-3. Dev Cell. 2007;13(1):15-28. Epub 2007/07/05. doi: 10.1016/j.devcel.2007.05.003. PubMed PMID: 17609107.
275. Moravec R, Conger KK, D'Souza R, Allison AB, Casanova JE. BRAG2/GEP100/IQSec1 interacts with clathrin and regulates alpha5beta1 integrin endocytosis through activation of ADP ribosylation factor 5 (Arf5). J Biol Chem. 2012;287(37):31138-47. Epub 2012/07/21. doi: 10.1074/jbc.M112.383117. PubMed PMID: 22815487; PubMed Central PMCID: PMC3438945.
276. Eskova A, Knapp B, Matelska D, Reusing S, Arjonen A, Lisauskas T, et al. An RNAi screen identifies KIF15 as a novel regulator of the endocytic trafficking of integrin. J Cell Sci. 2014;127(Pt 11):2433-47. Epub 2014/03/25. doi: 10.1242/jcs.137281. PubMed PMID: 24659801.
277. Finkielstein CV, Capelluto DG. Disabled-2: A modular scaffold protein with multifaceted functions in signaling. BioEssays. 2016;38 Suppl 1:S45-55. Epub 2016/07/16. doi: 10.1002/bies.201670907. PubMed PMID: 27417122.
278. Westcott JM, Prechtl AM, Maine EA, Dang TT, Esparza MA, Sun H, et al. An epigenetically distinct breast cancer cell subpopulation promotes collective invasion. J Clin Invest. 2015;125(5):1927-43. Epub 2015/04/07. doi: 10.1172/jci77767. PubMed PMID: 25844900; PubMed Central PMCID: PMC4463195.
279. Morishige M, Hashimoto S, Ogawa E, Toda Y, Kotani H, Hirose M, et al. GEP100 links epidermal growth factor receptor signalling to Arf6 activation to induce breast cancer invasion. Nat Cell Biol. 2008;10(1):85-92. Epub 2007/12/18. doi: 10.1038/ncb1672. PubMed PMID: 18084281.

280. Mai A, Muharram G, Barrow-McGee R, Baghirov H, Rantala J, Kermorgant S, et al. Distinct c-Met activation mechanisms induce cell rounding or invasion through pathways involving integrins, RhoA and HIP1. J Cell Sci. 2014;127(Pt 9):1938-52. Epub 2014/05/03. doi: 10.1242/jcs.140657. PubMed PMID: 24790222.
281. Li L, Liu DX, Zhang N, Liang Q, Feng J, Yao M, et al. SHON, a novel secreted protein, regulates epithelial-mesenchymal transition through transforming growth factor-beta signaling in human breast cancer cells. Int J Cancer. 2015;136(6):1285-95. Epub 2014/08/02. doi: 10.1002/ijc.29110. PubMed PMID: 25082541.
282. Caswell PT, Norman JC. Integrin trafficking and the control of cell migration. Traffic. 2006;7(1):14-21. Epub 2006/02/01. doi: 10.1111/j.1600-0854.2005.00362.x. PubMed PMID: 16445683.
283. Mendoza P, Díaz J, Silva P, Torres VA. Rab5 activation as a tumor cell migration switch. Small GTPases. 2014;5:e28195. doi: 10.4161/sgtp.28195. PubMed PMID: PMC4114561.
284. Frittoli E, Palamidessi A, Marighetti P, Confalonieri S, Bianchi F, Malinverno C, et al. A RAB5/RAB4 recycling circuitry induces a proteolytic invasive program and promotes tumor dissemination. J Cell Biol. 2014;206(2):307-28. Epub 2014/07/23. doi: 10.1083/jcb.201403127. PubMed PMID: 25049275; PubMed Central PMCID: PMC4107781.
285. Lindsay AJ, Hendrick AG, Cantalupo G, Senic-Matuglia F, Goud B, Bucci C, et al. Rab coupling protein (RCP), a novel Rab4 and Rab11 effector protein. J Biol Chem. 2002;277(14):12190-9. Epub 2002/01/12. doi: 10.1074/jbc.M108665200. PubMed PMID: 11786538.
286. Jacquemet G, Green DM, Bridgewater RE, von Kriegsheim A, Humphries MJ, Norman JC, et al. RCP-driven alpha5beta1 recycling suppresses Rac and promotes RhoA activity via the RacGAP1-IQGAP1 complex. J Cell Biol. 2013;202(6):917-35. Epub 2013/09/11. doi: 10.1083/jcb.201302041. PubMed PMID: 24019536; PubMed Central PMCID: PMC3776348.
287. Zhang J, Liu X, Datta A, Govindarajan K, Tam WL, Han J, et al. RCP is a human breast cancer-promoting gene with Ras-activating function. J Clin Invest. 2009;119(8):2171-83. Epub 2009/07/22. doi: 10.1172/jci37622. PubMed PMID: 19620787; PubMed Central PMCID: PMC2719918.
288. Cox D, Berg JS, Cammer M, Chinegwundoh JO, Dale BM, Cheney RE, et al. Myosin X is a downstream effector of PI(3)K during phagocytosis. Nat Cell Biol. 2002;4(7):469-77. Epub 2002/06/11. doi: 10.1038/ncb805. PubMed PMID: 12055636.
289. Arjonen A, Kaukonen R, Mattila E, Rouhi P, Hognas G, Sihto H, et al. Mutant p53-associated myosin-X upregulation promotes breast cancer invasion and metastasis. J Clin Invest. 2014;124(3):1069-82. Epub 2014/02/04. doi: 10.1172/jci67280. PubMed PMID: 24487586; PubMed Central PMCID: PMC3934176.
290. Zhang H, Berg JS, Li Z, Wang Y, Lang P, Sousa AD, et al. Myosin-X provides a motor-based link between integrins and the cytoskeleton. Nat Cell Biol. 2004;6(6):523-31. Epub 2004/05/25. doi: 10.1038/ncb1136. PubMed PMID: 15156152.
291. Cao R, Chen J, Zhang X, Zhai Y, Qing X, Xing W, et al. Elevated expression of myosin X in tumours contributes to breast cancer aggressiveness and metastasis. Br J Cancer. 2014;111(3):539-50. Epub 2014/06/13. doi: 10.1038/bjc.2014.298. PubMed PMID: 24921915; PubMed Central PMCID: PMC4119973.
292. Makowska Katarzyna A, Hughes Ruth E, White Kathryn J, Wells Claire M, Peckham M. Specific Myosins Control Actin Organization, Cell Morphology, and Migration in Prostate Cancer Cells. Cell Reports. 2015;13(10):2118-25. doi: 10.1016/j.celrep.2015.11.012. PubMed PMID: PMC4688110.
293. Siebert M, Sidransky E, Westbroek W. Glucocerebrosidase is shaking up the synucleinopathies. Brain. 2014;137(Pt 5):1304-22. Epub 2014/02/18. doi: 10.1093/brain/awu002. PubMed PMID: 24531622; PubMed Central PMCID: PMCPMC3999712.
294. Bondar C, Ormazabal M, Crivaro A, Ferreyra-Compagnucci M, Delpino MV, Rozenfeld PA, et al. Osteocyte Alterations Induce Osteoclastogenesis in an In Vitro Model of Gaucher Disease. Int J Mol Sci. 2017;18(1). Epub 2017/01/19. doi: 10.3390/ijms18010112. PubMed PMID: 28098793; PubMed Central PMCID: PMCPMC5297746.
295. Zhou X, Huang Z, Yang H, Jiang Y, Wei W, Li Q, et al. beta-Glucosidase inhibition sensitizes breast cancer to chemotherapy. Biomed Pharmacother. 2017;91:504-9. Epub 2017/05/10. doi: 10.1016/j.biopha.2017.04.113. PubMed PMID: 28478274.
296. Alcalay RN, Levy OA, Waters CC, Fahn S, Ford B, Kuo SH, et al. Glucocerebrosidase activity in Parkinson's disease with and without GBA mutations. Brain. 2015;138(Pt 9):2648-58. Epub 2015/06/29. doi: 10.1093/brain/awv179. PubMed PMID: 26117366; PubMed Central PMCID: PMCPMC4564023.

297. Pchelina S, Emelyanov A, Baydakova G, Andoskin P, Senkevich K, Nikolaev M, et al. Oligomeric alpha-synuclein and glucocerebrosidase activity levels in GBA-associated Parkinson's disease. Neurosci Lett. 2017;636:70-6. Epub 2016/10/27. doi: 10.1016/j.neulet.2016.10.039. PubMed PMID: 27780739.
298. Lee JH, McBrayer MK, Wolfe DM, Haslett LJ, Kumar A, Sato Y, et al. Presenilin 1 Maintains Lysosomal Ca2+ Homeostasis via TRPML1 by Regulating vATPase-Mediated Lysosome Acidification. Cell Reports. 2015;12(9):1430-44. doi: 10.1016/j.celrep.2015.07.050. PubMed PMID: WOS:000360574200008.
299. Cai Y, An SS, Kim S. Mutations in presenilin 2 and its implications in Alzheimer's disease and other dementia-associated disorders. Clinical interventions in aging. 2015;10:1163-72. Epub 2015/07/24. doi: 10.2147/cia.s85808. PubMed PMID: 26203236; PubMed Central PMCID: PMCPMC4507455.
300. Guerra E, Cimadamore A, Simeone P, Vacca G, Lattanzio R, Botti G, et al. p53, cathepsin D, Bcl-2 are joint prognostic indicators of breast cancer metastatic spreading. BMC Cancer. 2016;16:649. Epub 2016/08/20. doi: 10.1186/s12885-016-2713-3. PubMed PMID: 27538498; PubMed Central PMCID: PMCPMC4991058.
301. Caswell PT, Spence HJ, Parsons M, White DP, Clark K, Cheng KW, et al. Rab25 associates with alpha5beta1 integrin to promote invasive migration in 3D microenvironments. Dev Cell. 2007;13(4):496-510. Epub 2007/10/11. doi: 10.1016/j.devcel.2007.08.012. PubMed PMID: 17925226.
302. Agola JO, Jim PA, Ward HH, Basuray S, Wandinger-Ness A. Rab GTPases as regulators of endocytosis, targets of disease and therapeutic opportunities. Clin Genet. 2011;80(4):305-18. Epub 2011/06/10. doi: 10.1111/j.1399-0004.2011.01724.x. PubMed PMID: 21651512; PubMed Central PMCID: PMC3187864.
303. Saha SK, Choi HY, Kim BW, Dayem AA, Yang GM, Kim KS, et al. KRT19 directly interacts with beta-catenin/RAC1 complex to regulate NUMB-dependent NOTCH signaling pathway and breast cancer properties. Oncogene. 2016. Epub 2016/06/28. doi: 10.1038/onc.2016.221. PubMed PMID: 27345400.
304. Lu X, Yan C, Huang Y, Shi D, Fu Z, Qiu J, et al. Mouse double minute 2 (MDM2) upregulates Snail expression and induces epithelial-to-mesenchymal transition in breast cancer cells in vitro and in vivo. Oncotarget. 2016. Epub 2016/05/18. doi: 10.18632/oncotarget.9287. PubMed PMID: 27184007.
305. Aerbajinai W, Liu L, Zhu J, Kumkhaek C, Chin K, Rodgers GP. Glia Maturation Factor-gamma Regulates Monocyte Migration through Modulation of beta1-Integrin. J Biol Chem. 2016;291(16):8549-64. Epub 2016/02/21. doi: 10.1074/jbc.M115.674200. PubMed PMID: 26895964; PubMed Central PMCID: PMC4861427.

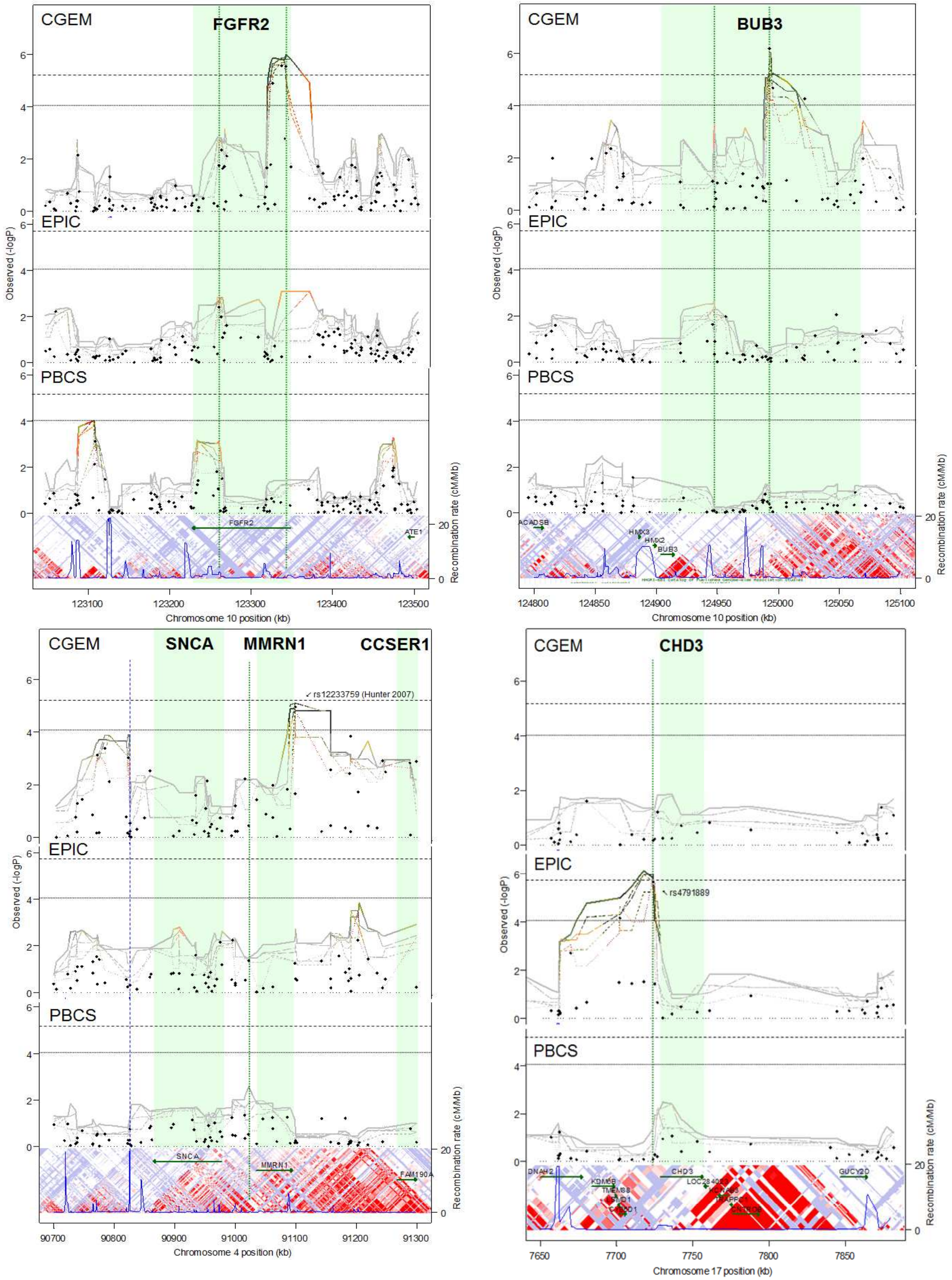

Fig E: Top and replicated genes Top: DGEM, Center: EPIC, bottom: PBCS

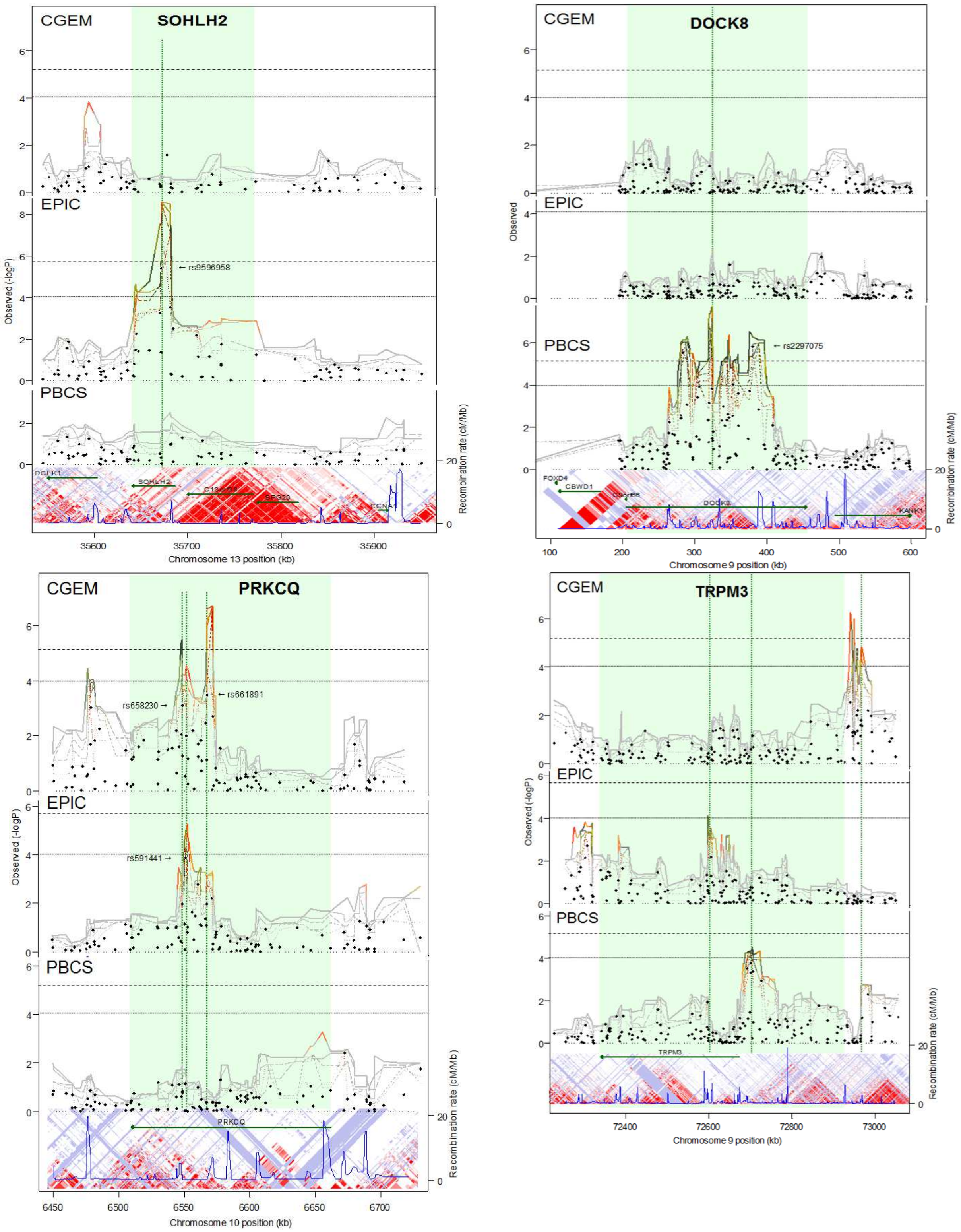

Fig E: Top and replicated genes (continued)

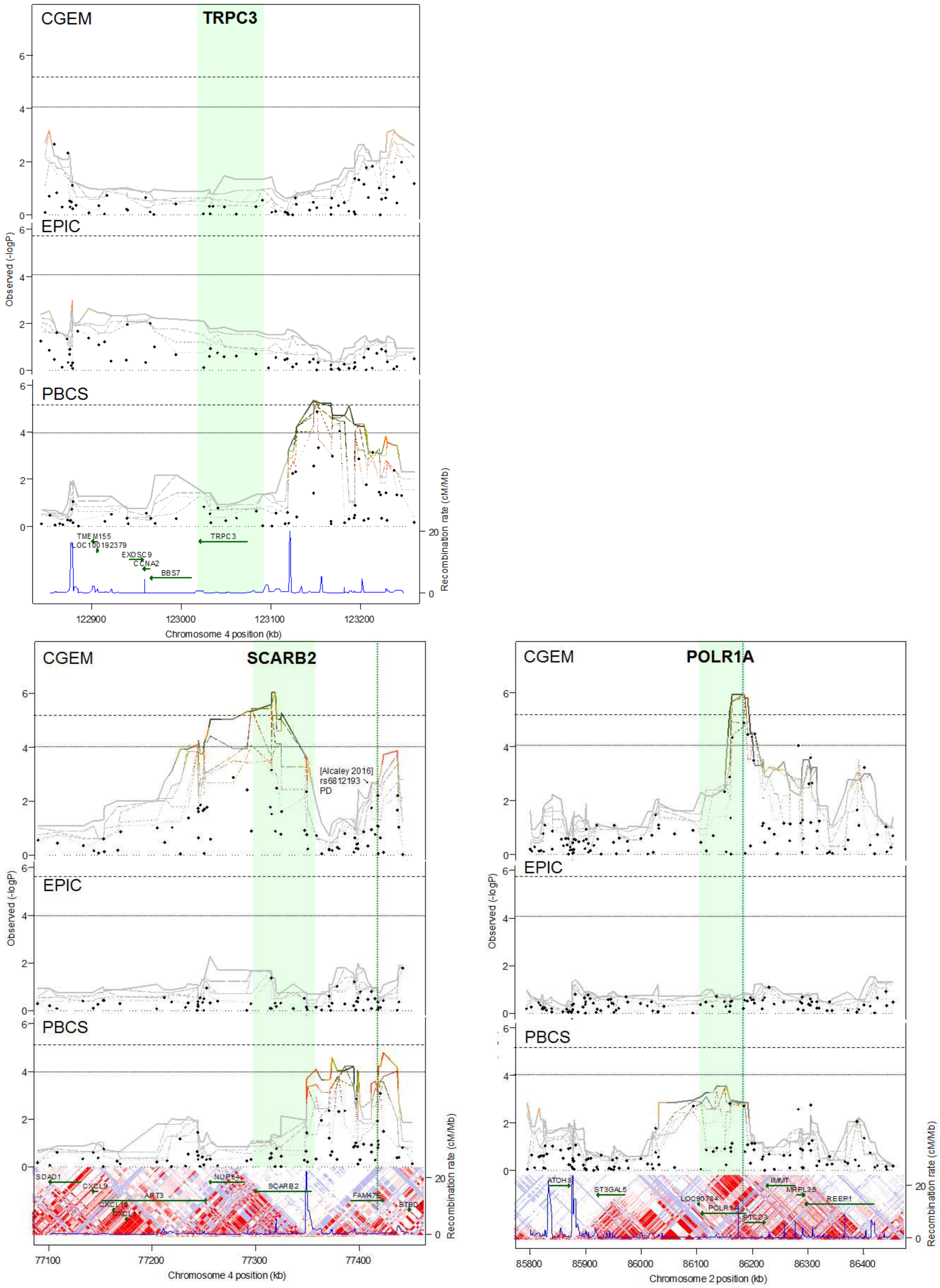

Fig E: Top and replicated genes (continued)

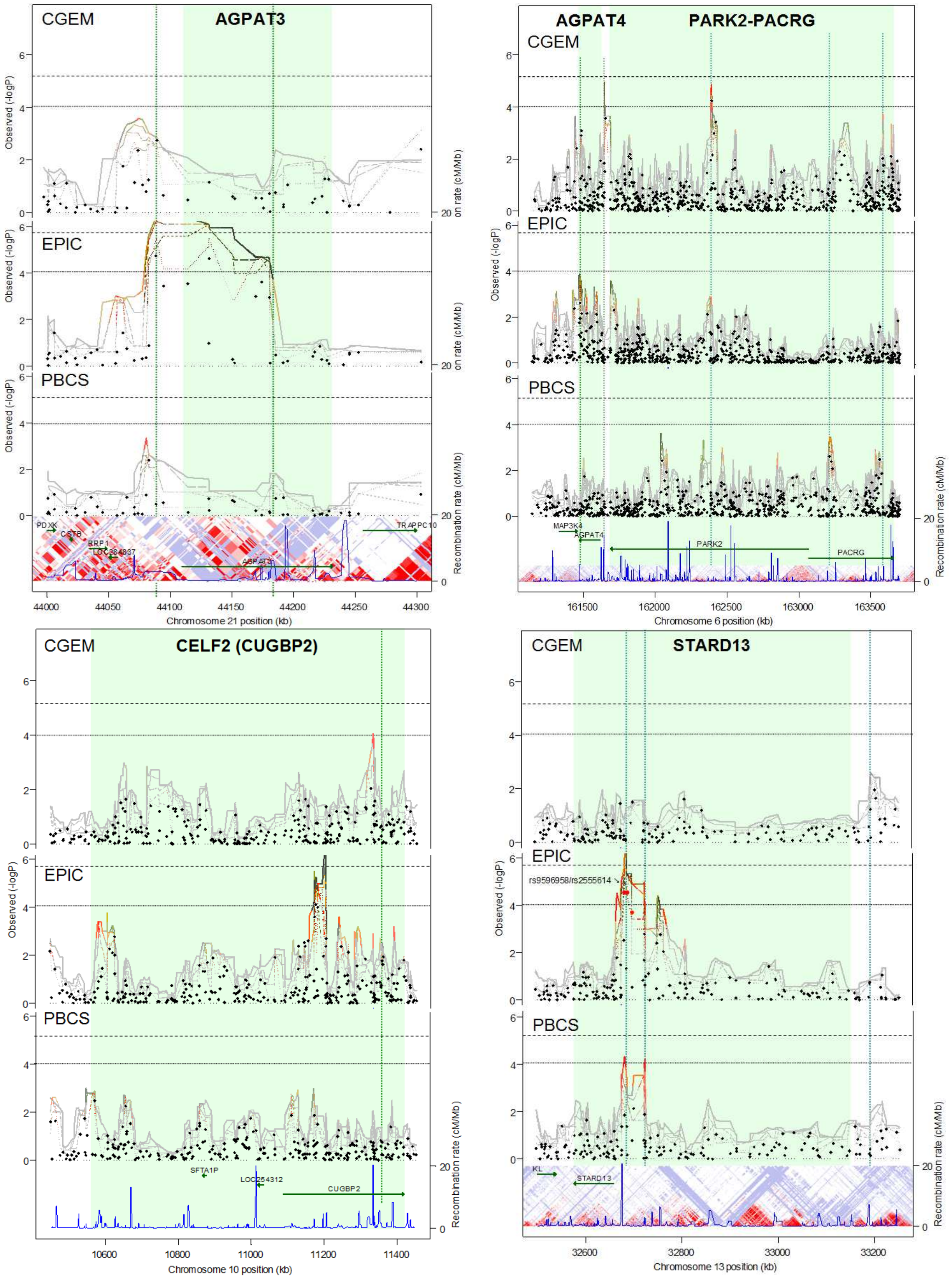

Fig E: Top and replicated genes (continued)

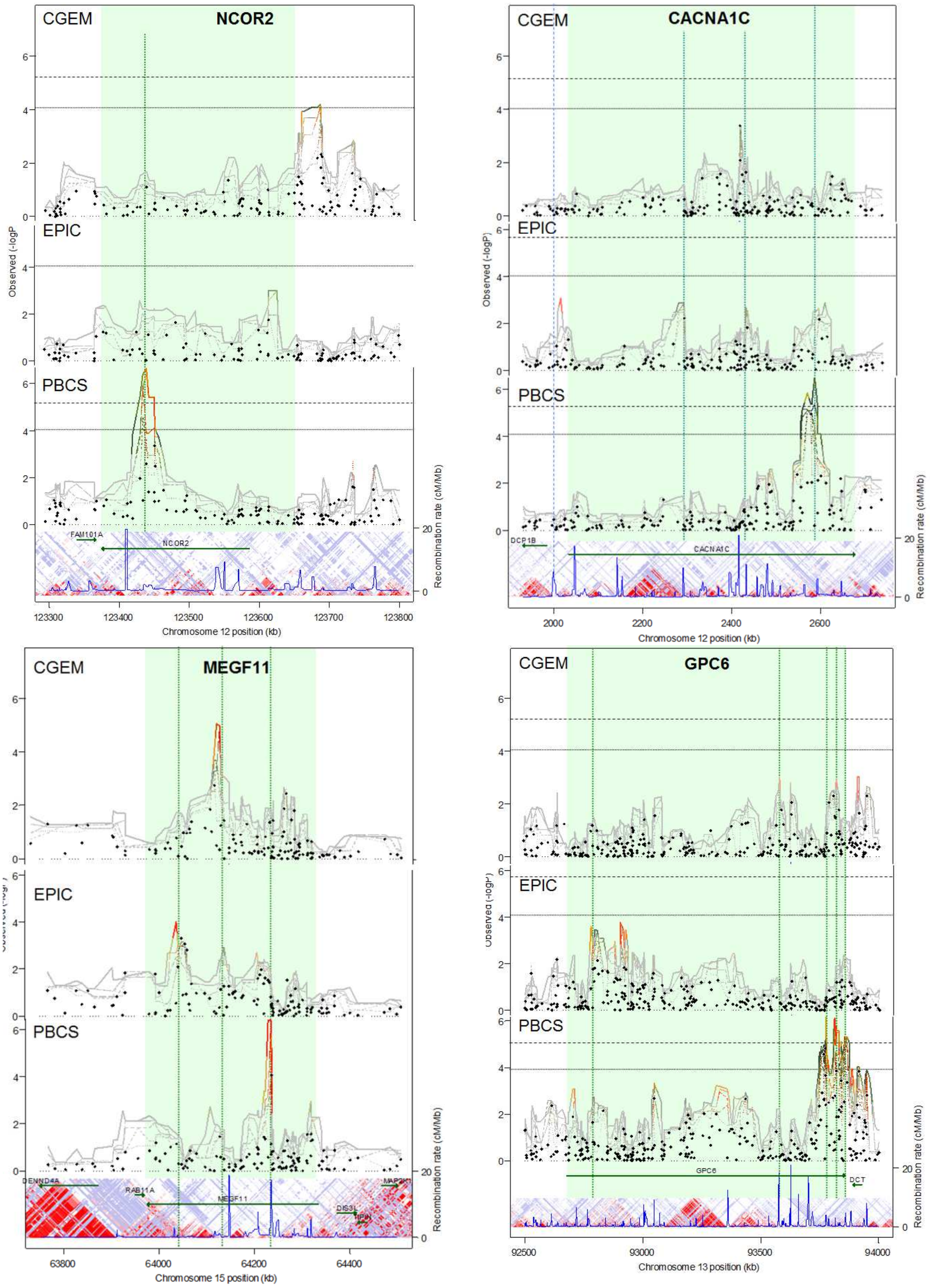

Fig E: Top and replicated genes (continued)

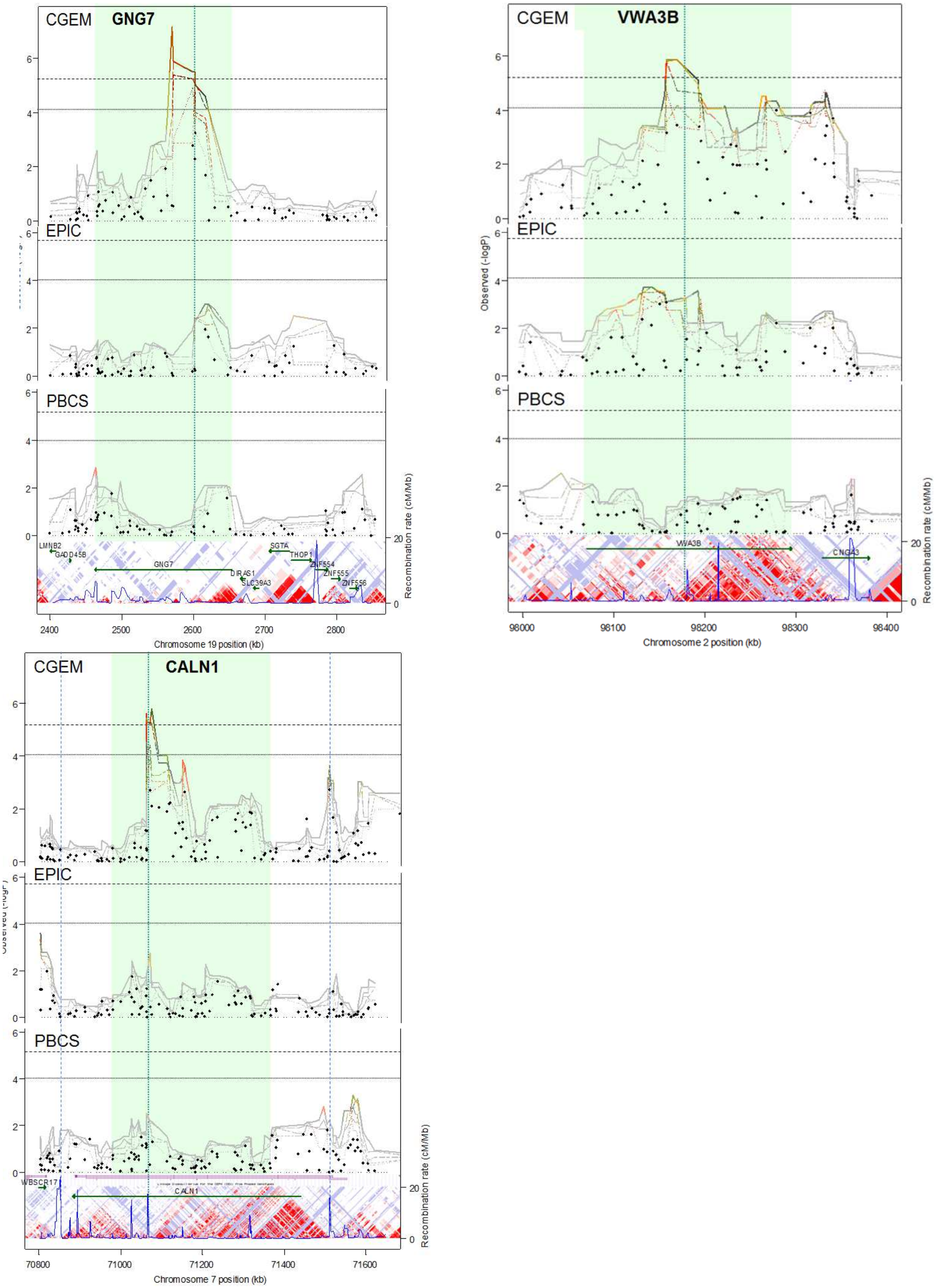

Fig E: Top and replicated genes (continued)

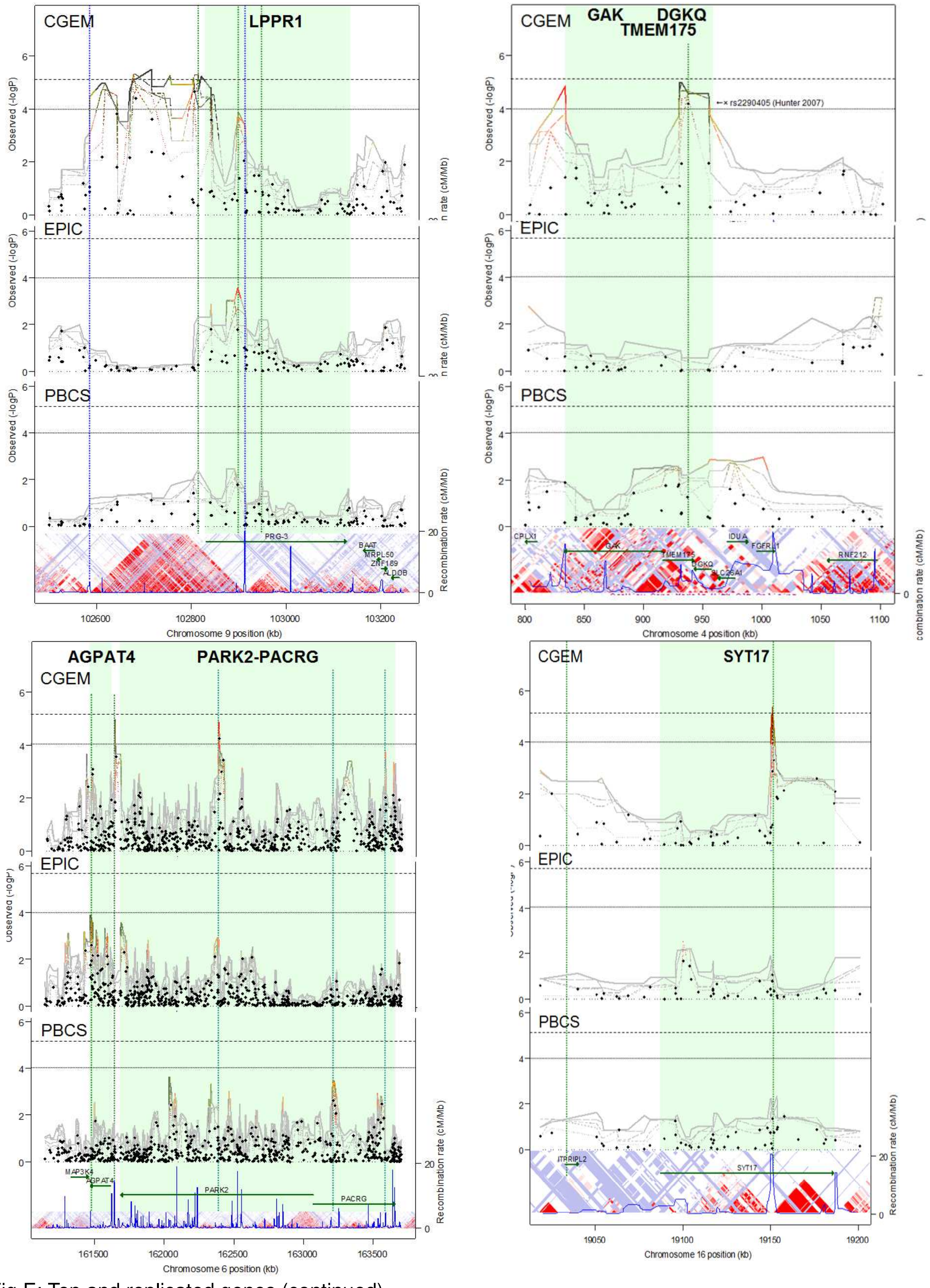

Fig E: Top and replicated genes (continued)

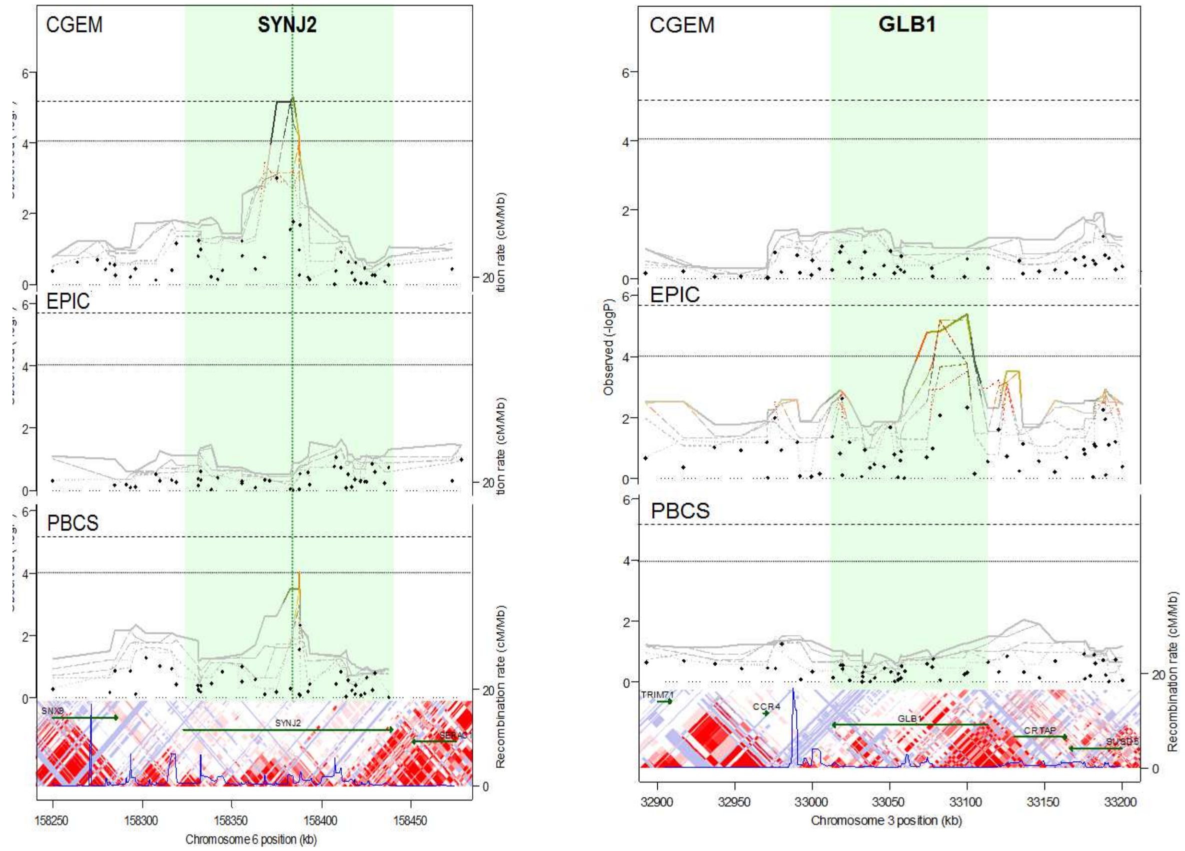

Fig E: Top and replicated genes (continued)

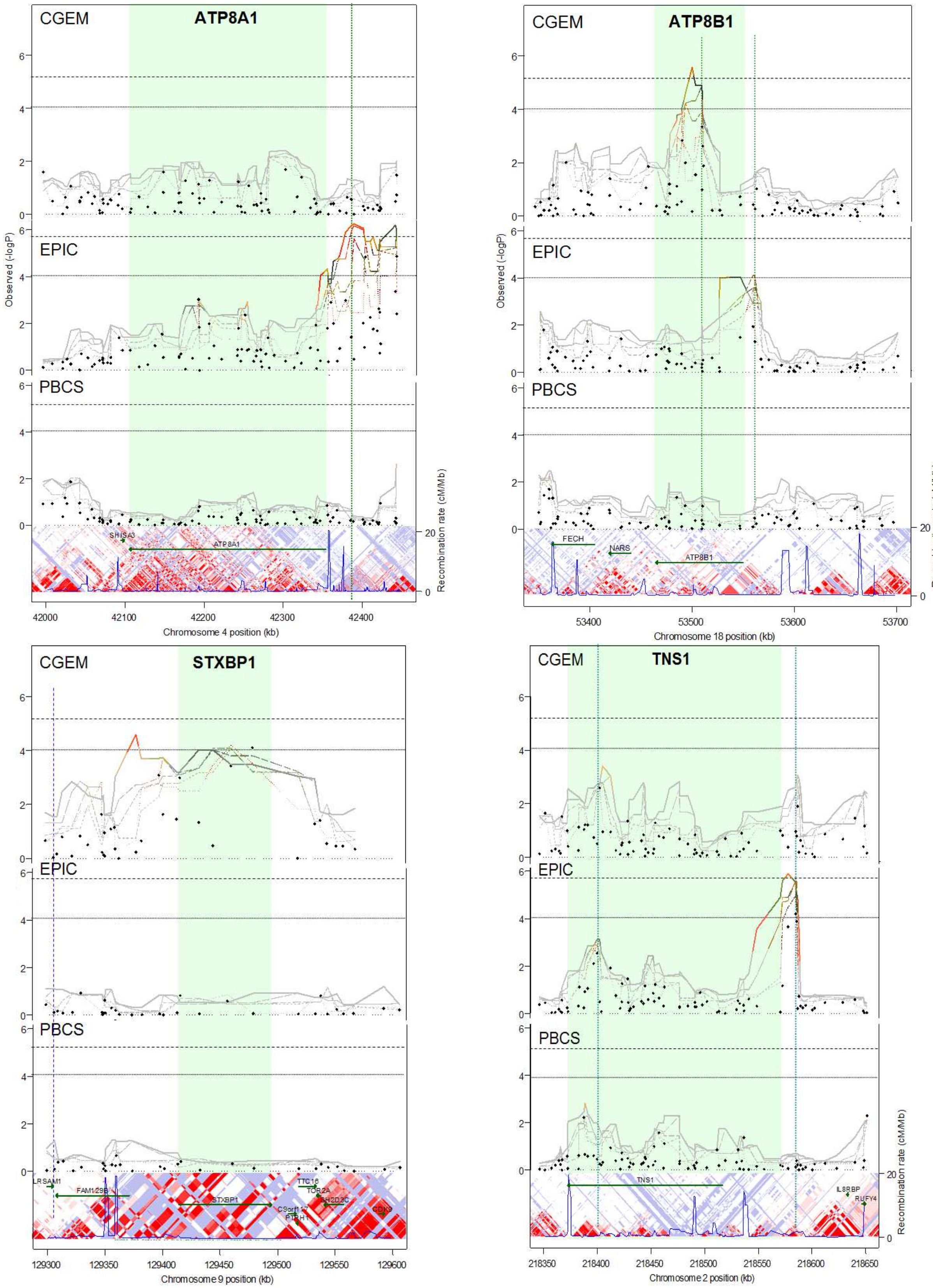

Fig E: Top and replicated genes (continued)

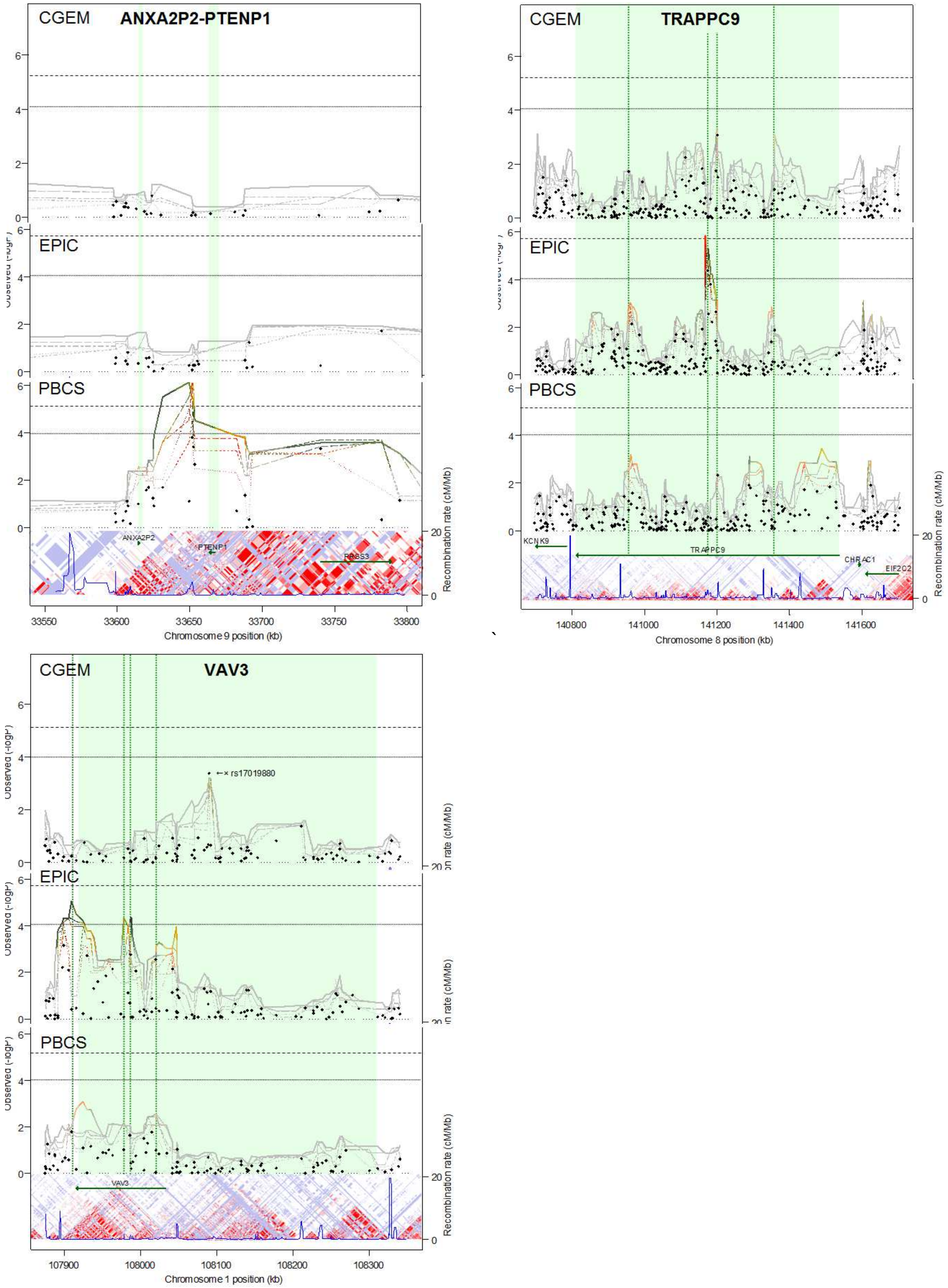

Fig E: Top and replicated genes (continued)

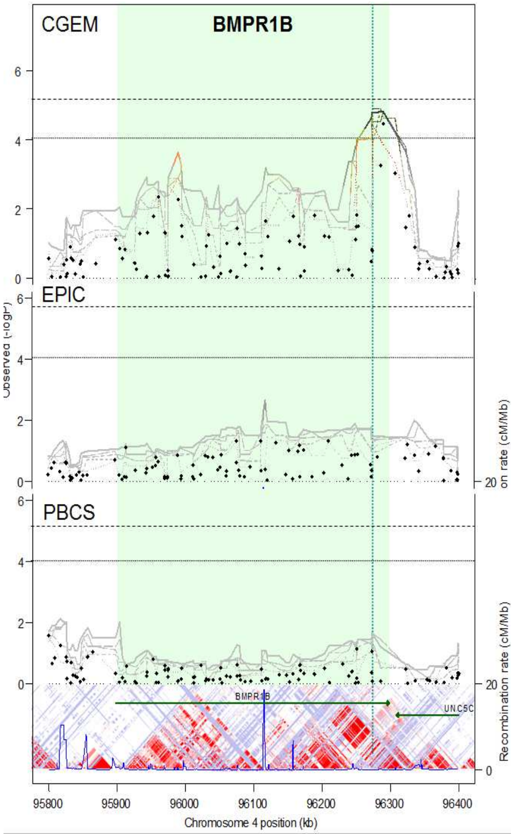

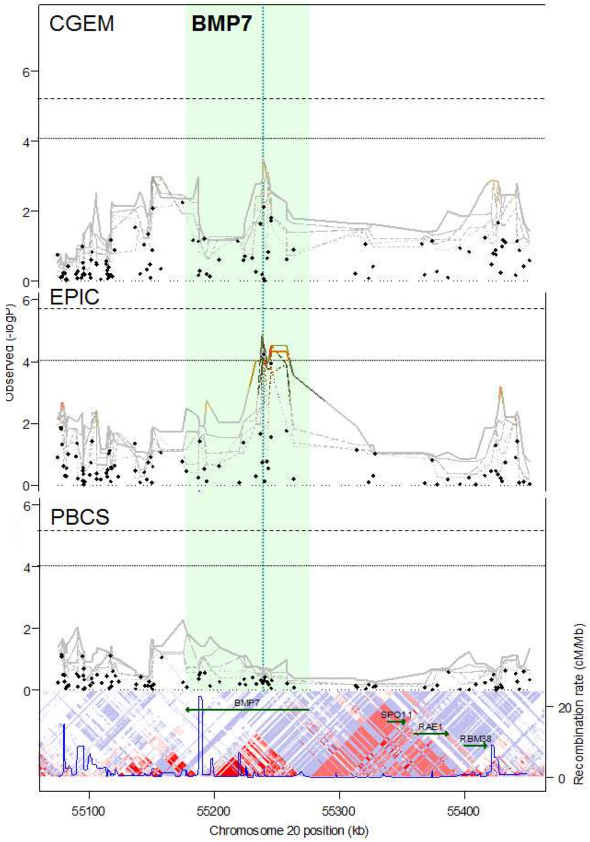

Fig E: Top and replicated genes (continued)

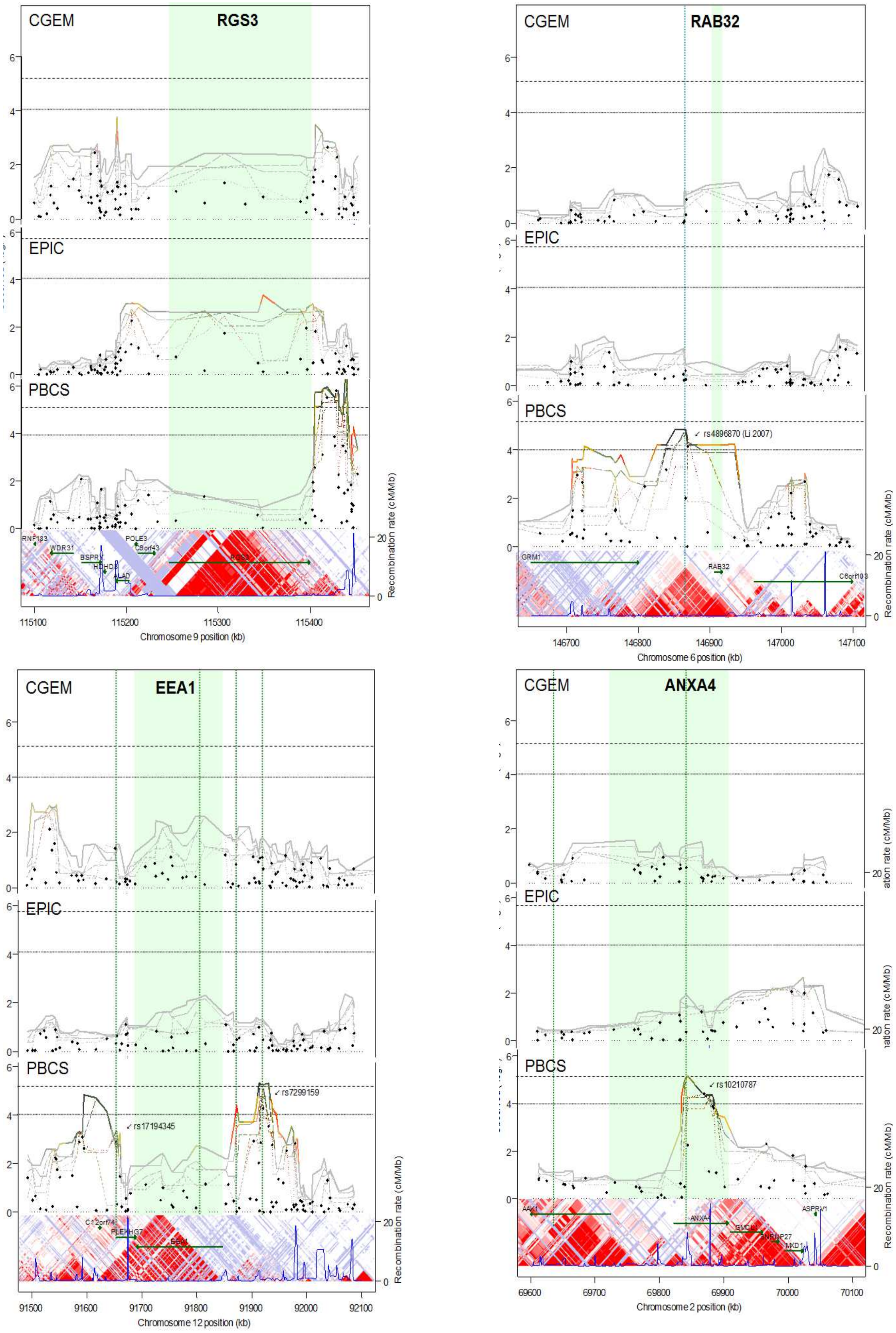

Fig E: Top and replicated genes (continued)

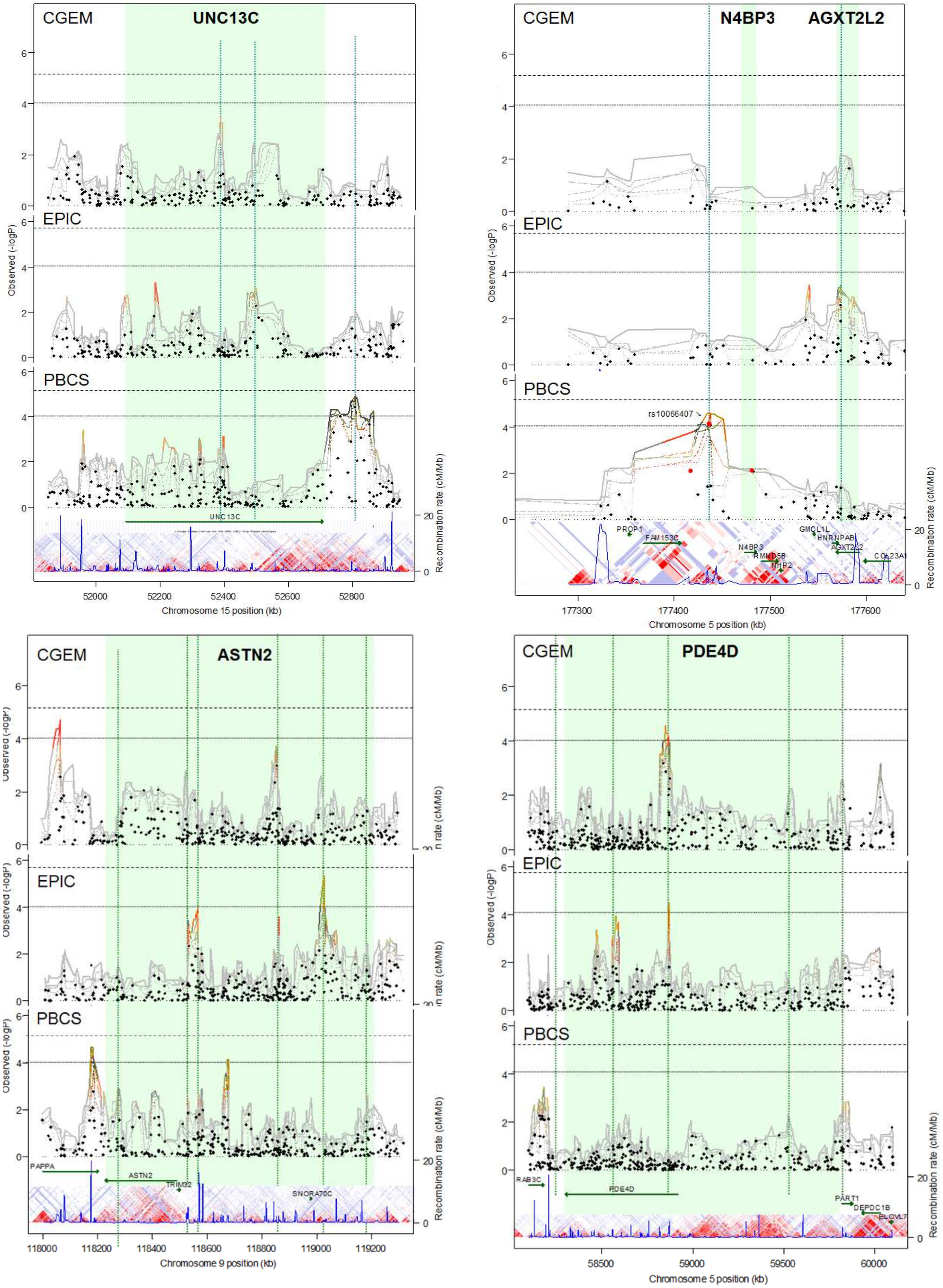

Fig E: Top and replicated genes (continued)

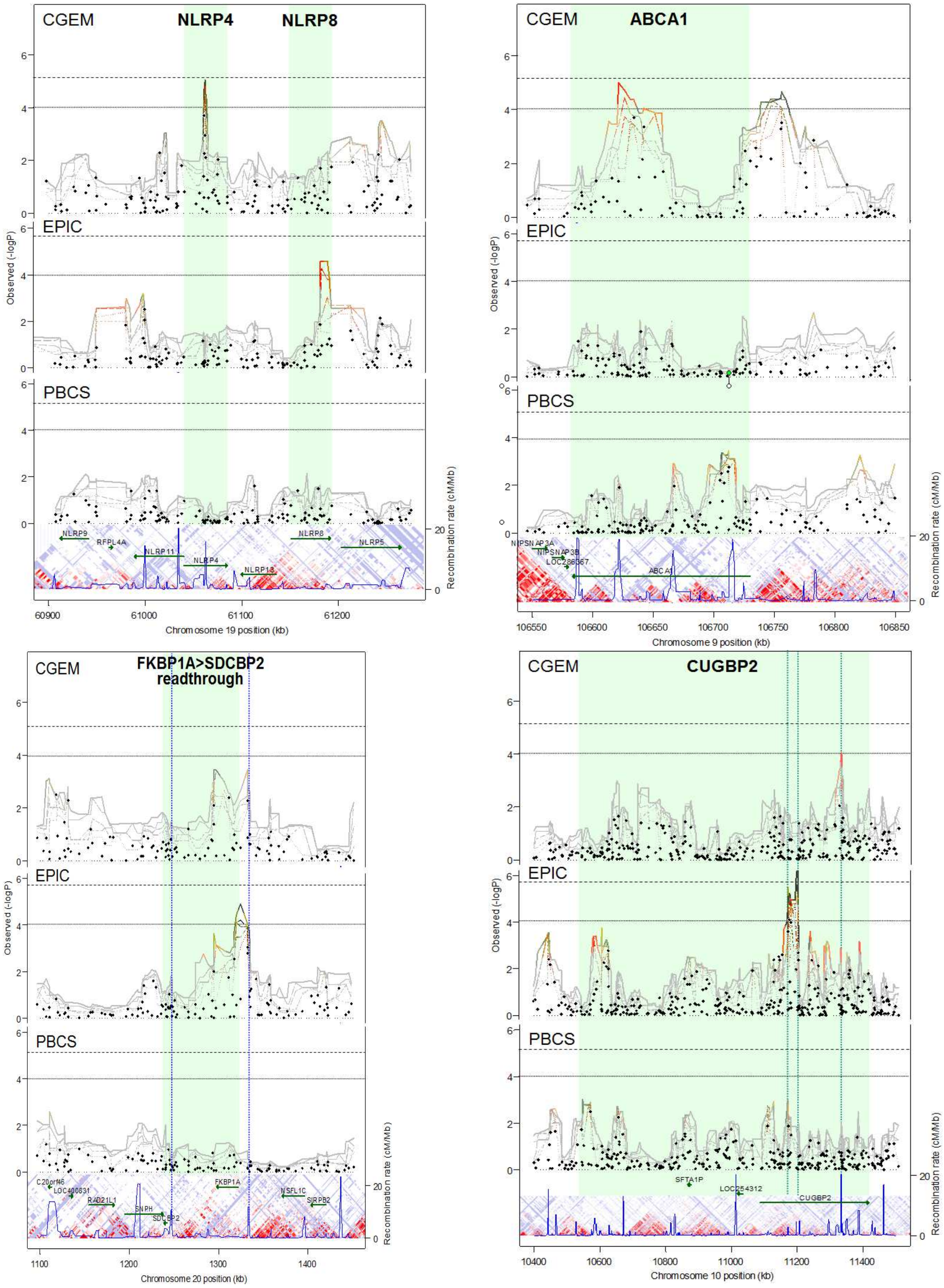

Fig E: Top and replicated genes (continued)
